# High-resolution prosthetic hearing with a soft auditory brainstem implant in macaques

**DOI:** 10.1038/s41551-025-01378-9

**Published:** 2025-04-18

**Authors:** Alix Trouillet, Emilie Revol, Florent-Valéry Coen, Florian Fallegger, Aurélie Chanthany, Maude Delacombaz, Laurine Kolly, Ivan Furfaro, Florian Lanz, Vivek Kanumuri, Victor Adenis, Alejandro Garcia-Chavez, M. Christian Brown, Lukas Anschuetz, Jocelyne Bloch, Daniel J. Lee, Stéphanie P. Lacour

**Affiliations:** 1 Laboratory for Soft Bioelectronic Interfaces, Neuro X Institute, School of Engineering, Ecole Polytechnique Fédérale de Lausanne EPFL, Geneva, Switzerland; 2 Department of Neuroscience, Platform of Translational Neuroscience, University of Fribourg, Fribourg, Switzerland; 3 Eaton-Peabody Laboratories, Department of Otolaryngology, Massachusetts Eye and Ear and Harvard Medical School, Boston, MA USA; 4 Department of Otorhinolaryngology, Lausanne University Hospital, Lausanne, Switzerland; 5 Department of Clinical Neuroscience, Lausanne University Hospital (CHUV), Lausanne, Switzerland; 6 NeuroRestore, Defitech Center for Interventional Neurotherapies, EPFL/CHUV/UNIL, Lausanne, Switzerland; 7 Neuro X Institute, School of Life Sciences, Ecole Polytechnique Fédérale de Lausanne EPFL, Geneva, Switzerland

**Keywords:** Biomedical engineering, Peripheral neuropathies, Auditory system

## Abstract

Individuals with compromised cochlear nerves are ineligible for cochlear implants and instead rely on auditory brainstem implants (ABIs). Most users of ABIs experience sound awareness, which aids in lip reading, yet not speech intelligibility. Here we engineered a dual-site (brainstem and cortex) implantable system, scaled to macaque anatomy, for the analysis of auditory perception evoked by electrical stimulation of the cochlear nucleus. A soft multichannel ABI, fabricated using thin-film processing, provided high-resolution auditory percepts, with spatially distinct stimulation sites eliciting cortical responses akin to frequency-specific tuning. Behavioural responses collected over several months were sufficiently precise to distinguish stimulations from adjacent channels. Soft multichannel ABIs may aid the rehabilitation of individuals with profound hearing loss who are ineligible for cochlear implants.

## Abstract (web summary)

Soft auditory brainstem implant in the cochlear nucleus of macaque evokes auditory-like perceptions with high resolution and tonotopic specificity.

## Main

Deafness is a debilitating sensory disorder that impacts individuals of every age and substantially affects their quality of life. According to the World Health Organization, 700 million people will require hearing rehabilitation by 2050. Indeed, the recent explosion of noise exposure in recreational settings puts 1.1 billion young people, aged 12–35 years old, at risk of permanent sensorineural hearing loss. Other factors leading to auditory impairment include genetic hearing loss as well as non-congenital factors such as infection, ototoxic medications, posterior fossa tumours and aging.

Among treatments to address sensorineural deafness and more generally in the field of neuroprostheses, cochlear implants have been a success story^1^. They provide meaningful sound perception to children and adults with unilateral or bilateral severe to profound hearing loss that do not benefit from amplification. Although complex auditory tasks such as musical appreciation are not accurately encoded by the cochlear implant^2^, most users experience open set speech recognition in quiet^3^. Modern CIs have multichannel arrays implanted into the cochlea, in which there is an orderly arrangement of neurons sensitive to different sound frequencies, a ‘tonotopic’ organization. Each cochlear implant electrode conveys information about a narrow band of frequencies.

A unique cohort of auditory implant candidates have hearing loss due to a compromised or absent cochlea or cochlear nerve. Most of these individuals have neurofibromatosis type 2 (NF2), a devastating genetic syndrome resulting in multiple brain and spinal cord tumours, including bilateral vestibular schwannomas. The incidence of NF2 is approximately 1:33,000 worldwide and is inherited as an autosomal dominant condition in 50% of individuals or a de novo pathogenic variant in the other 50%. Almost all individuals with NF2 develop adult-onset profound hearing loss due to the growth or treatment of vestibular schwannomas that damage the cochlear nerves^4^, rendering the cochlear implant ineffective in these cases. The auditory brainstem implant (ABI) was initially developed in the 1980s and approved in 2000 by the US Food and Drug Administration (FDA) for hearing rehabilitation in individuals with NF2. The ABI is a modified cochlear implant that bypasses the auditory periphery (the cochlea and cochlear nerve) and electrically stimulates the surface of the cochlear nucleus with a multichannel array^5–7^. The cochlear nucleus resides in the pontomedullary junction of the brainstem, has a curved and complex topography^8^, and is the first central relay station for all sound information originating in the ear^9^.

Similar to the cochlear implant, the ABI processor separates the acoustic spectrum into frequency bands that are matched to a grid of surface electrodes to generate tonotopic perception. However, unlike cochlear implant recipients, most ABI users experience sound awareness that aids in lip reading but only limited or negligible speech comprehension^10–12^. Moreover, a number of brainstem nuclei (such as the trigeminal, facial, glossopharyngeal) surround the cochlear nucleus and off-target effects often arise from unintended spread of electrical current^13^. Thus, deactivation of some ABI electrodes is necessary owing to side effects^12,14^ such as pain, facial twitching or dizziness^15^.

The cochlear implant is placed during an outpatient mastoidectomy surgery, and the electrode array is inserted through the round window to conform to the spiral form factor and tonotopically organized cochlea. ABI placement is much more complex and requires an invasive craniotomy approach to reach the posterior fossa and visualize the cranial nerves and brainstem. The CN is never directly visualized during surgery, necessitating the use of indirect anatomic landmarks and electrophysiology to ensure accurate positioning of the electrode array^16^. This ‘blind’ placement contributes to the ABI’s variable and modest outcomes^13,16^.

Accessing the curved cochlear nucleus without producing off-target effects is difficult with the clinical ABI, which has a stiff electrode array that prohibits good tissue contact^9^. In addition, stimulation patterns currently used in the clinic have been transposed directly from cochlear implant paradigms, hence stimulation of the tonotopic organization is not always optimal. There is a paucity of research that focuses on the development of ABI technologies as well as relevant chronic animal models to test hypotheses. Most studies utilize mouse models for cochlear nucleus stimulation^17–19^ and this may limit translation to clinical care in humans.

**Fig. 1:**
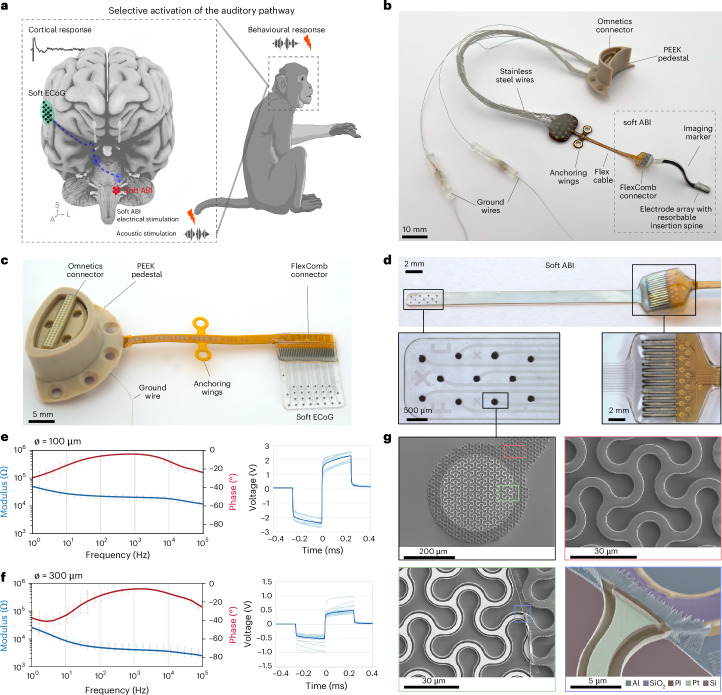
Experimental design and devices. **a**, Left: schematic of an NHP brain. The soft ABI is implanted on the cochlear nucleus; the soft ECoG records cortical responses from the auditory cortex. Right: NHP behavioural task in response to electrical or acoustic stimulation. Created with BioRender.com (https://BioRender.com/y29o453) and using a brain image from Adobe Stock. **b**, Photograph of the integrated soft ABI and its connectors: an Omnetics connector is embedded in a PEEK pedestal and connected to the soft ABI. An imaging marker and resorbable insertion spine are also visible. **c**, Integrated soft ECoG device: 29 electrodes (300-µm diameter) that record cortical signals. **d**, Close-up of the soft ABI: 11 electrodes (100-µm diameter). Right inset: FlexComb connector^22^. **e**,**f**, In vitro characterization^23^ of the soft ABI (**e**) and EcoG (**f**). Left: EIS measurement, where data points represent the average and bars represent standard errors for all electrodes. Right: VT in response to a 100-µA biphasic current pulse. The thick blue line represents the average and light lines represent individual electrodes. **g**, Scanning electron microscopy (SEM) images of micropatterned ABI electrodes and interconnects. Top left: image of one ABI electrode and associated interconnect. Red frame, close-up of an interconnect; green frame, close-up of the electrode. Bottom right: colourized SEM of the electrode and encapsulation of the platinum layer within the polyimide dielectric layers. Source data

On the basis of preliminary work showing the feasibility and potential of soft technology for cochlear nucleus stimulation in mice and human cadavers^9^, we developed a chronic, non-human primate (NHP) model that incorporates advanced electrode implant technology^9^. We propose an alternative to the existing clinical ABI by leveraging soft bioelectronic advances^9^ and NHP translational research. We engineered a dual-site (brainstem and cortex) implantable system, allowing stimulation and recording from the auditory pathway, using soft electrodes and elastic constructs of thin-film multilayers optimized for long-term use in vivo. Through read-outs both at the cortical (soft electrocorticography (ECoG) implant) and behavioural levels, we compared auditory stimulation vs electrical stimulation (Fig. 1a). We validated the soft bioelectronic system with brain electrophysiology and clinically relevant behavioural tasks over several months, which provides a feasible strategy towards improved central auditory prosthesis technology for clinical rehabilitation.

## Results

### Design of the experimental protocol

The NHP model integrates multiple components, each critical for assessing the efficacy of the hearing prosthesis (Fig. 1a). First, using advanced microfabrication techniques, we designed and manufactured two personalized and NHP-scaled implants: a soft multichannel ABI array to stimulate the cochlear nucleus (Fig. 1b,d) and a soft ECoG array to record the responses (auditory evoked potentials (AEPs)) from the auditory cortex (Fig. 1c). We also recorded auditory brainstem responses (ABRs), which are used clinically to evaluate acoustic hearing and ABI placement in humans. Then, we implemented a behavioural frequency discrimination task to assess the perceptual impact of electrical stimulation delivered via the soft ABI.

### Design of the chronic NHP-size soft ABI

The conformability of an implant to the curvilinear surface of the nervous system is governed by the choice of its materials and their geometry, which allow the computation of the bending energy of the implant. We used the elastocapillarity model developed previously^20^ to anticipate the geometry of an implant to wrap around a specific curvature around the cochlear nucleus. The brainstem anatomy in two NHPs was examined with histology ex vivo and structural imaging in vivo to quantify the morphology of the cochlear nucleus, including curvature and surface area (~3 mm (ref. ^2^)). These measurements indicated that an electrode array width of 2 mm and length of 2.7 cm was appropriate (that is, from the tip to the craniotomy exit). Using computed tomography (CT) and magnetic resonance imaging (MRI), completed by 3D reconstructions, and the findings from a previous study^9^, we determined that the gap between the cochlear nucleus and the temporal bone could fit an electrode array of a maximum thickness of 200 μm. In addition, assuming that the kirigami pattern introduces negligible stiffening to the silicone rubber membrane that carries the implant, a minimal radius of the cochlear nucleus of 3 mm, a Young’s modulus of the silicone of 1 MPa and a cerebrospinal fluid surface tension of *γ* = 61 mN m^−1^, the maximal thickness of the implant should be 170 μm. As a result, we designed an ABI of 150 μm thickness, hosting 11 electrodes, distributed over 0.96 mm × 2.64 mm, each with a diameter of 100 µm, accounting for the patterning resolution of the soft neurotechnology^9^ (Fig. 1b,d and Supplementary Fig. 1a,b).

### Microfabrication of the soft ABI

The ABI was engineered with stretchable interconnects, fully embedded within a 150-μm-thick layer of silicone rubber (Fig. 1d). This design ensured critical mechanical compliance and preserved electrical integrity, essential for sustained biocompatibility and durability in vivo. The electrical tracks consisted of a microstructured stack of polyimide/platinum/polyimide (PI/Pt/PI) films^9^. Integral to the design, the replicated motifs within the stack impart stretchability to the interconnects. The resulting low bending stiffness ensured conformability to curved anatomical structures such as the cochlear nucleus^8,9^. To promote long-term reliability of the implant, the conductive and dielectric layers were patterned independently so that the platinum film was fully embedded within the polyimide sheath (Fig. 1g). The electrode contacts were coated with a soft platinum-silicone composite^21^ that supports efficient charge injection properties and provides a soft interface with the tissue (Supplementary Fig. 2). A FlexComb^22^ interconnection system that transitions from the soft ABI to a flexible printed circuit board (PCB) completed the electrode implant. After fabrication, the soft ABI was characterized in vitro using electrochemical impedance spectroscopy (EIS) and chronopotentiometry (Fig. 1e). The voltage transient (VT), measured in response to a 100-µA biphasic current pulse, shows an average access voltage of 1.96 V, corresponding to a resistive drop of 19 kΩ. This value captures the contribution of the resistive components of the system, with the micropatterned tracks being the main contributor. Soft ABI maximal charge injection capacity (CIC), measured in vitro^23^, was found to be ~1,524 µC cm^−2^, within the range of CIC for plain platinum electrodes (typically between 150 and 5,570 μC cm^−2^)^24^. Similar post-fabrication characterization was performed for the ECoG (Fig. 1f).

### Implantation and intra-operative validation

A posterior fossa surgical approach was utilized for placement of the soft ABI array in two animals (monkey L and monkey G). Under general anaesthesia, we identified cranial landmarks to indicate the position of the transverse and sigmoid sinus, and then completed a 3D exoscope-assisted keyhole retrosigmoid craniectomy to expose the dura mater. The dura was incised and cerebrospinal fluid (CSF) was drained. Rigid Hopkins rod telescopes (0 and 30°) coupled to a 2D 4 K video camera provided an endoscopic-assisted wide-angle view of the posterior fossa and cerebellopontine angle through the keyhole craniotomy. We identified relevant anatomical landmarks including the cerebellar peduncle, choroid plexus and cranial nerves VII, VIII, IX, X and XI (Supplementary Fig. 3a). The root entry zones of the IX (glossopharyngeal) nerve and choroid plexus were observed. The soft ABI array was placed under endoscopic visualization into the lateral recess of the 4th ventricle and foramen of Luschka to approximate the surface of the dorsal subdivision of the cochlear nucleus. Surgical insertion was facilitated by grasping a bioresorbable spine bonded to the back of the array (Supplementary Fig. 3b). Specifically designed for this purpose, the poly(vinyl alcohol) (PVA) hydrogel spine dissolved after ~15 min at 37 °C.

**Fig. 2:**
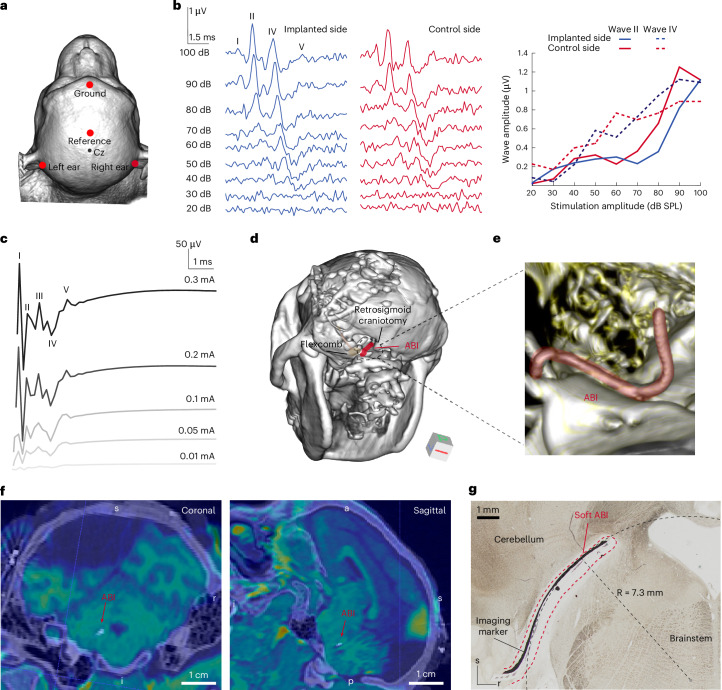
Soft ABI placement and validation both intra- and post-operatively. **a**, Recording setup for ABRs and eABRs, showing the position of ground and reference electrodes. **b**, Post-surgery ABRs for both implanted (left) and control (right) sides. Traces show responses from 20 to 100 dB SPL. Graph displays wave II and IV amplitude depending on stimulation amplitude. **c**, Intra-operative eABR responses to ABI monopolar stimulation (elec1) at increasing stimulation amplitude. Waves I, II, III, IV and V are labelled. The first 1 ms of the trace was removed because of contamination with the stimulus artefact. **d**, 3D reconstruction based on CT images of NHP cranium. The FlexComb connector^22^ is visible on CT and highlighted in light brown. The ABI imaging marker is highlighted in red as it enters the posterior fossa craniotomy. Orientation is indicated. **e**, Magnified view of CT 3D reconstruction focusing on the electrode array (in red). The reconstruction shows the flexibility of the soft array. **f**, Co-registered CT and MRI images taken post surgery, showing the ABI artefact from the imaging marker. The bone is shown in purple colour and soft tissue of the brainstem is shown in green colour in two different imaging planes (sagittal, coronal; a, anterior; p, posterior; l, left; r, right; s, superior; i, inferior). **g**, Histological brainstem coronal cross-section showing the electrode array in place after 17 months of implantation. The black line is the imaging marker, and the full pad is delimited by the dashed red line. This image reveals the in situ conformability of the soft ABI at the surface of the brainstem. The radius curvature of the tip of the soft ABI in this image was measured as 7.3 mm. Source data

Accurate placement of the soft ABI array was confirmed by monitoring electrically evoked far field responses, called electrically evoked auditory brainstem responses (eABRs, Fig. 2c and Supplementary Fig. 4a), for each electrode in the monopolar configuration. Facial electromyography responses (EMG) were co-monitored with eABRs to assess side effects and adjust ABI placement. Once verified, the implant position was secured using a muscle graft.

Next, the flex PCB cable was fixed to the bone at the exit of the craniotomy using the integrated anchoring wings (Fig. 1b), artificial dura mater and free muscle transfer. Then, fibrin glue was applied to ensure hermeticity and avoid CSF leak (Supplementary Fig. 3c). The connector was secured in a customized transdermal head-stage pedestal and screwed to the skull (Supplementary Fig. 5). Auditory function was measured by monitoring post-surgical acoustic ABRs. We recorded ABR thresholds of 40–50 dB SPL (sound pressure level) (Fig. 2a,b), which were similar to pre-surgery ABR thresholds.

### Soft ABI localization and stability over time

The position of the soft ABI array was confirmed following surgery on CT and MRI images (Fig. 2d–f). A dedicated radio-opaque marker integrated on the array surface (Fig. 1b) enabled the precise localization of the tip of the array. By co-registering CT with MR images, we confirmed the placement at the surface of the cochlear nucleus (Fig. 2f). Magnified views of 3D CT imaging reconstruction confirm conformability of the device in situ (Fig. 2e). Stability of the implant position was assessed by serial CT. In monkey L, no quantifiable displacement was observed over the 9-month implantation. At 17 months after implantation and following perfusion and sectioning, histology showed the soft ABI still in place (Fig. 2g). The radius of curvature of the soft ABI conforming to the surface of the cochlear nucleus was 7.3 mm. In monkey G, however, we observed a migration of the soft ABI intra-operatively.

### Characterization of cortical responses to electrical stimulation

**Fig. 3:**
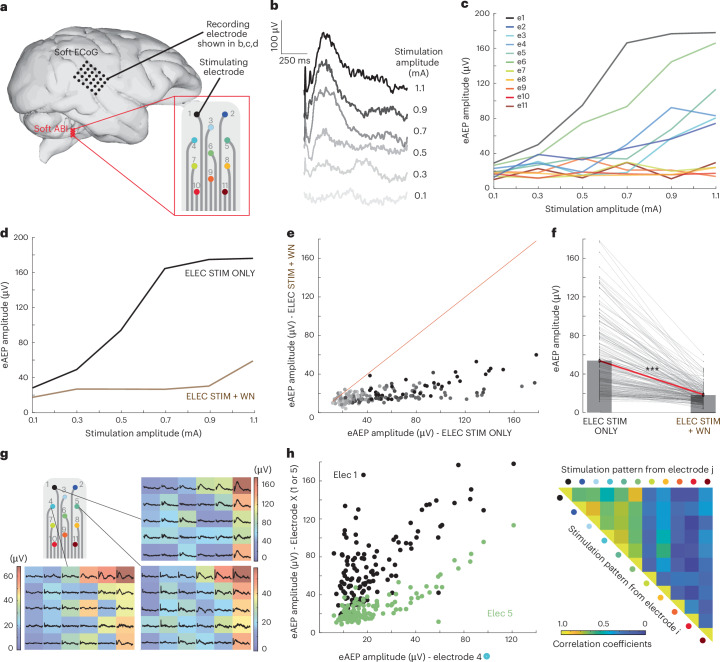
Characterization of cortical responses to soft ABI stimulation. **a**, Experimental design schematic. ECoG dimension: 300-µm-diameter electrodes, 1.43 mm *x*-direction pitch, 1.33 mm *y*-direction pitch. **b**, eAEP recorded by the ECoG (recording channel 1) when ABI electrode e1 is stimulated (current: 0.1–1.1 mA; pulse width: 300 µs). **c**, Recruitment curves for each ABI electrode as a function of stimulation level (recording channel 1). In this experiment, e7 to e11 were unresponsive in the stimulated range. **d**, Electrical stimuli were presented to ABI electrode e1 with (STIM + WN, brown curve) and without (STIM ONLY, black curve) acoustic white noise (WN) in the background; example of response from 1 representative ECoG recording channel. **e**, Each dot represents a response from one ECoG channel (peak to peak amplitude, µV) generated by a given electrical stimulation current delivered by ABI e1 (same grey scale as **b**) with and without white noise in the background. The distribution of the responses with and without white noise does not follow the *x* = *y* line (red). **f**, Mean of all responses that is significantly different when white noise is applied in the background (one-tailed paired *t*-test, ***P < 0.001). **g**, Colourmaps representing the activity at the surface of the auditory cortex (eAEPs peak to peak amplitude, µV) depending on which electrode is stimulated (at a given stimulation amplitude, in this example, 0.9 mA). Each trace represents the average over 1 s. **h**, Linear correlation between electrodes e4 and e5, while electrodes e1 and e4 are not correlated. Each dot represents an electrode of the ECoG array at a given stimulation amplitude (single pulse ranging from 0.1 mA to 1.1 mA). Right: table showing the mean correlation coefficients from all ECoG recording channels for each ABI electrode pair. Panel **a** was created using brain reconstruction from refs. ^43,44^. Source data

In both subjects (monkey L chronically, monkey G intra-operatively), we recorded cortical responses to sound (auditory evoked potentials, AEPs) and to ABI electrical stimulation (electrical auditory evoked potentials, eAEPs). Before ABI surgery, each animal was implanted with a subdural soft ECoG array (Fig. 1c and Supplementary Fig. 1c,d) positioned on the contralateral (right) auditory cortex (Supplementary Fig. 6a–c) and secured on the skull (Supplementary Fig. 6d). The geometry and position of the ECoG were anticipated using MRI imaging and 3D reconstruction of the animal’s brain (Supplementary Fig. 6e,f). We performed cortical mapping (300-µm-diameter recording electrodes, 1.43 mm *x*-direction pitch, 1.33 mm *y*-direction pitch, Supplementary Fig. 1c) using single-pulse monopolar stimulation (0.01–2 mA, 100–600 µs pulse width) of different electrodes of the soft ABI (Fig. 3a and Supplementary Fig. 1a). Latency of the responses were used to distinguish evoked potentials from earlier stimulation artefacts (Supplementary Fig. 4b). Activation thresholds, based on eAEP amplitudes, were as low as 0.3 mA (Fig. 3b). eAEP amplitudes ranged from 50 to 170 µV, comparable to AEPs (20–90 µV). The dynamic range of the eAEP, defined from threshold to saturation, suggests that electrical stimulation delivered by the ABI may modulate the intensity of the neuroprosthetic percept (Fig. 3a–c) similarly to auditory cues (Supplementary Fig. 7b,c).

To confirm the auditory nature of the eAEP response, we introduced competing acoustic noise during electrical stimulation. The mean eAEP responses across the ECoG dropped from 50.9 µV to 17.34 µV when adding white noise (Fig. 3d–f, *P* < 0.001) similarly to the masking of natural acoustic responses (12.9 µV for acoustic stimulation only, compared with 7.77 µV for acoustic stimulation and white noise) (Supplementary Fig. 7d–f, *P* < 0.001). The reduction in amplitude, which caused recruitment curves to shift towards higher thresholds (Fig. 3d–f), confirmed the auditory nature of the eAEP response.

### Neuroprosthetic tonotopy of the soft ABI

We compared the spatial patterns of evoked cortical responses under both acoustic and electrical stimulation. Distinct activity patterns on the auditory cortex were induced by electrical stimulation through different ABI electrodes (Fig. 3g), similarly to the tonotopic maps triggered with acoustic stimulation (Supplementary Fig. 7g). For example, ABI electrode pairs, such as e1–e5, produced correlated ECoG signals, whereas other pairs, for example e1–e4, resulted in markedly different patterns (Fig. 3h). These patterns of correlation also appeared with acoustic stimulation: frequencies of 1 kHz and 2 kHz were highly correlated, whereas 1 kHz and 5 kHz induced substantially different cortical activations (Supplementary Fig. 7h). We confirmed that these patterns of correlation were not statistically different when stimulating electrically or acoustically (Supplementary Fig. 7i) but both were statistically different from a random pattern (Supplementary Fig. 7i, *P* < 0.0001). These findings highlight the specificity of ABI stimulation and demonstrate the ability of closely spaced electrodes (e1 and e4 are 0.88 mm apart, Supplementary Fig. 1a) to elicit distinct activity patterns. Interestingly, correlation was not proportional either to electrode physical spacing or acoustic spectral spacing. Recordings made directly from ABI electrodes showed that acoustic stimulation at various frequencies elicited spatially segregated auditory evoked cochlear nucleus responses (AECNRs) (Supplementary Fig. 8).

### Behavioural responses to electrical stimulation

**Fig. 4:**
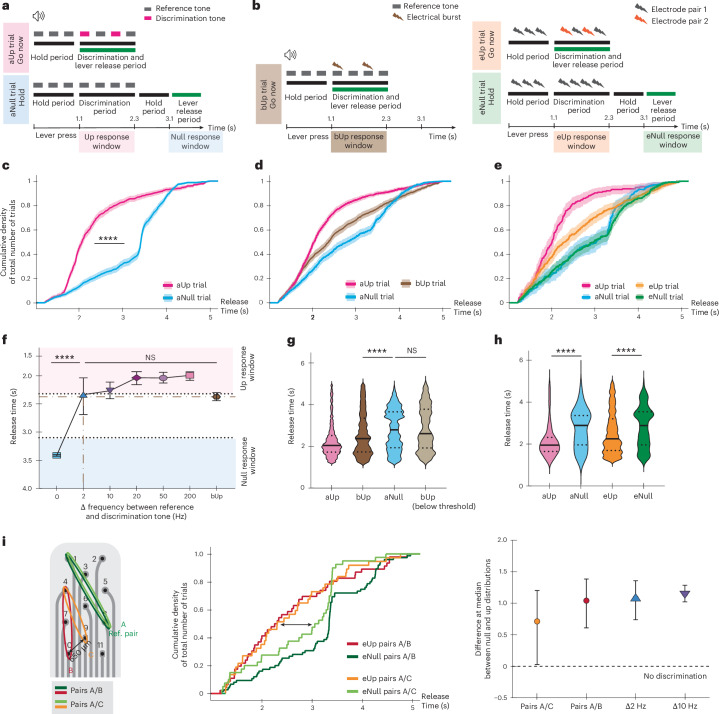
Behavioural evaluation of the soft ABI. **a**,**b**, Designs and lever release periods for the aTask (**a**), and the bTask (left) and eTask (right) (**b**). **c**–**e**, Lever release distributions with 95% confidence interval (CI; shaded area) for the aUp and aNull trials (**c**); release times are statistically different (Mann–Whitney unpaired two-tailed *t*-test, ****P < 0.0001) (*n*_aUp_ = 747, *n*_aNull_ = 846); for the bUp trials (**d**); distribution is distinct from the aNull one (Mann–Whitney unpaired two-tailed *t*-test, *P* < 0.0001) and tends towards the aUp distribution (*n*_aNull_ = 726, *n*_aUp_ = 1102, *n*_bUp_ = 879); and for the eTask with auditory trials for comparison (**e**) (*n*_eUp_ = 372, *n*_eNull_ = 447, *n*_aUp_ = 228, *n*_aNull_ = 226). **f**, Lever release median and 95% CI for different types of aUp trial (*n*_aUp_200Hz_ = 104, *n*_aUp_50Hz_ = 117, *n*_aUp_20Hz_ = 126, *n*_aUp_10Hz_ = 120, *n*_aUp_2Hz_ = 68). Release time for a difference of |2 Hz| is statistically different from that of aNull trials (Mann–Whitney unpaired two-tailed *t*-test, ****P < 0.0001), while bUp trials are not (Mann–Whitney unpaired two-tailed *t*-test, NS). **g**, bUp trials (above threshold stimulation) are statistically different from aNull trials (Mann–Whitney unpaired two-tailed *t*-test, ****P < 0.0001), while bUp trials (below threshold stimulation) are not (Mann–Whitney unpaired two-tailed *t*-test, NS) (*n*_bUp_belowThreshold_ = 126). Median (centre line) and quartiles (dotted lines) are displayed. **h**, Lever release times are statistically different for eUp and eNull trials (Mann–Whitney unpaired two-tailed *t*-test, ****P < 0.0001), similarly to auditory trials. Median and quartiles are displayed. **i**, Performance from the electrical trials (middle panel) involving ABI pairs A/B (8-1/10-4) displays greater distribution differences than for ABI pairs A/C (8-1/9-4). The difference at the median (right panel, with 95% CI) between the respective eUp and eNull distribution is closer to 0 for pairs A/C (pair A/B *n*_eNull_ = 75, *n*_eUp_ = 46; pair A/C *n*_eNull_ = 40, *n*_eUp_ = 37). Source data

We tested whether electrical stimulation was associated with perceptual saliency in one subject trained on an acoustic task (monkey L, Supplementary Fig. 9). The behavioural task was based on a Go/NoGo paradigm^25^ with positive reinforcement (Fig. 4a). The animal was trained to initiate a trial by pressing and holding a lever. Reference and discrimination stimuli were then presented. When the animal heard two stimuli that it perceived as different (up trial – go now), the correct response was to release the lever within a short time frame (1.1 s and 2.3 s). However, when the two stimuli were the same (null trial – hold) a correct response occurred with a long release time (3.1 s). Performance on the tasks was evaluated by comparing the distribution profiles for different trial types (see Methods). There were three different tasks tested: an acoustic task (‘aTask’; Fig. 4a), an electrically biased acoustic task (‘bTask’; Fig. 4b, left) and an electrical task (‘eTask’; Fig. 4b, right).

First, we trained the animal to an acoustic frequency discrimination task, allowing a determination of baseline discrimination (aTask; Fig. 4a). To obtain a reward, the animal had to discriminate between a reference tone and a discrimination tone (different frequencies = ‘aUp’ trial, same frequency = ‘aNull’ trial). This acoustic training was performed over several months before ABI implantation, to ensure robust understanding of the task and a reliable control condition. The animal learned to discriminate between different frequencies (Fig. 4c, *P* < 0.0001), down to a 2-Hz frequency variation (Fig. 4f, *P* < 0.0001).

Following placement of the ABI, an electrically biased acoustic trial was introduced within the discrimination task (bTask; Fig. 4b, left) which was randomly presented between the acoustic trials. Specifically, we superimposed ABI electrical stimulation to the discrimination tone of an acoustic aNull trial (during the discrimination period only); both electrical and acoustic stimulations were synchronized. The animal was able to discriminate this condition ‘bUp’ from an acoustic aNull trial (Fig. 4d,g, *P* < 0.0001), similarly to the acoustic aUp condition. The response distributions for these electrical trials were not significantly different from responses to an acoustic frequency difference of 2 Hz between discrimination and reference tones. This suggests that the ABI-induced percept shift was similar to such a difference (Fig. 4f). The control trials, using subthreshold ABI stimulation, were not significantly different from the acoustic aNull trials presented during the same session (Fig. 4g). These results suggest that the ABI stimulation modulated the acoustic perception of the animal and was indeed perceived as an acoustic cue.

To further investigate the spatial resolution of the ABI, we assessed the animal’s ability to discriminate stimulation applied between two different electrode pairs of the soft ABI (electrical trials, eTask; Fig. 4b, right). We tested several pairs as probe and reference stimuli in behavioural sessions where both purely acoustic and purely electrical trials were randomly interleaved. The animal successfully performed this eTask without the need for a learning phase (Fig. 4e). These results confirm that stimulation from two electrodes of the soft ABI was perceived differently by the animal. When the stimulation was purely electrical, the animal achieved a performance comparable to the acoustic task aTask (Fig. 4h, *P* < 0.0001).

Finally, we compared the performance between different pairs of neighbouring electrodes of the soft ABI. As the distance between electrode pairs decreased (Supplementary Fig. 1a and Fig. 4i), the discrimination performance was reduced, while it remained above discrimination levels. A similar trend was noted with acoustic stimulation, specifically in the discrimination of Δ*f* = 10 Hz and Δ*f* = 2 Hz in acoustic stimuli (Δ*f* denotes frequency difference; Fig. 4i).

Our observations indicated neither electromyographic (EMG) activation of cranial or facial musculature, nor any signs of pain or changes in feeding or vocalization behaviours during activation of the 11 ABI electrodes within the parameters set for behavioural or electrophysiologic testing. In active conditions within the task, the animal consistently demonstrated readiness and willingness to press the lever to initiate trials, showing no aversion or fear of receiving stimulation through the soft ABI. This observation is considerable given that the types of trial were randomly presented, meaning the animal could not anticipate the specific nature of the upcoming trial.

## Discussion

We developed a soft auditory neuroprosthesis that was surgically implanted onto the brainstem of NHP and tested over a period of ~5 months. Our soft ABI successfully elicited robust auditory brainstem and cortical responses in two animals, and demonstrated effective behavioural auditory discrimination in one of them. Moreover, we report cochlear nucleus recordings (AECNRs) collected via the soft ABI^26^ in response to acoustic stimulation, along with cortical recordings (eAEPs) in response to electrical stimulation of the cochlear nucleus. The miniaturized soft implant, combined with this NHP model, represents a series of notable advances in the field of auditory neuroprostheses.

First, we leveraged microfabrication techniques to engineer a soft ABI, scaled to the NHP anatomy, that conforms to the contoured surface of the brainstem while minimizing its overall footprint. This soft multichannel ABI array (containing 11 electrodes, each 0.1 mm in diameter) is 2 mm wide and 0.15 mm thick. In comparison, the clinically approved ABI includes 21 electrode contacts, 0.7–0.6 mm in diameter, embedded within a silicone array of 0.6-mm thickness. The use of macroscopic components and manual manufacturing processes considerably limits the potential for miniaturization and personalization of traditional neural implants. By contrast, high-density microelectrode arrays leverage manufacturing techniques from the semiconductor industry, allowing for the precise patterning of micron-scale structures over large areas with consistent accuracy and high throughput. These methods also enable scalable customization of implants since they do not depend on dedicated, single-use parts such as plastic moulds.

In addition, the small microelectrode diameters used here allow for a high local charge density, leading to focal stimulation that activates neurons that are likely to be located directly below the electrode. Such focal stimulation, while beneficial for increasing the tonotopy of cochlear nucleus responses, could on the other hand lead to charge density so high that it might cause tissue damage. The soft ABI maximal CIC of ~1,524 µC cm^−2^, measured in vitro, falls within the range of CIC for plain platinum electrodes^24^. However, this limit might not completely reflect in vivo reality, as the measurement was performed in vitro using −0.6 V as safety limit, which, according to ref. ^24^, might be subject to variation with electrode size and might not be the same for platinum particles vs plain platinum. In addition, micro-electrodes in general might not fully follow the charge/phase and charge density relationship, as depicted in the Shannon equation used to define the biological safe limit of electrical stimulation. For this reason, it is difficult to directly assess tissue damage using micro-electrodes from charge density or maximal CIC. Interestingly, in our case, we did not notice apparent tissue damage: hearing function was preserved throughout the entire experiment and there was no apparent biological damage as evidenced by the lack of observable tissue necrosis around the implantation site (Fig. 2g).

In contrast to our design, the clinical ABI does not conform to the curvature of the human cochlear nucleus (3-mm radius^8,9^). This lack of conformability may be a contributing factor to the limited efficacy of the ABI in humans. During ABI fitting sessions, clinicians often have to deactivate most of the electrodes because patients are experiencing pain, discomfort or other non-auditory side effects^27^. The extent of deactivation varies from patient to patient, with a couple to even all electrodes being turned off^28^. A 10-year follow-up study of 24 patients^12^ reported an average of 13 electrodes being deactivated. Nevertheless, the number of active electrodes is a critical feature for accessing the tonotopic representation at the surface of the cochlear nucleus. Achieving specific and distinct frequency discrimination and interpreting complex stimulation sequences are contingent on the device’s design tailored to the patient’s needs. The study of ref. ^12^ indicates that maintaining at least 11 active electrodes is essential for ensuring reasonable word detection capabilities^25^.

The mechanical and physical properties of the soft ABI can, in principle, improve the contact between the cochlear nucleus and electrodes, thereby reducing electrical stimulation thresholds and off-target activation^9^. In clinical settings, the average eABR thresholds^14^ are ~22.3 nC, as reported in 17 patients^29^ fitted with ABIs having 0.6-mm-diameter electrodes. For the soft ABI, the range of eAEP activation thresholds is ~60–210 nC, with electrodes six times smaller than clinical electrodes. To test whether the soft microfabricated ABI achieves better tissue contact compared with standard clinical implants, we compared outcomes of soft ABI stimulation with those seen in patients, such as side effects and frequency perception. Side effects were not observed at stimulation levels that produced auditory behavioural responses, whereas in the human clinical ABI, side effects are common. Together, these suggest the absence of pain or discomfort during soft ABI stimulation, allowing the full resolution of the implant to be utilized without the need to deactivate any electrodes. However, we cannot exclude the possibility of other kinds of non-auditory sensations potentially evoked by the soft ABI stimulation.

The soft ABI is MRI compatible owing to its minimal footprint combined with very thin metal layers^30^. These features create only minimal imaging artefacts, offering an advantage for clinical translation in terms of safety and negligible tissue masking. It is especially pertinent for ABI candidates, who are commonly individuals with NF2 tumours, requiring regular MRI scans for tumour monitoring. In the context of this study, precise localization of the ABI post implantation was crucial. The inclusion of an imaging marker on the surface of the implant enabled the creation of sufficient imaging artefacts for its clear visualization in vivo.

Our evaluation of the neuroprosthetic outcomes in the NHP model involved a systematic assessment of the auditory pathway’s responses under both natural and electrical stimulation. The NHP model offers advantages: the cochlear nucleus is larger than that of common rodent models so that a soft ABI with a higher number of electrodes can be used. It enabled prolonged training and testing periods. Importantly, the anatomical and physiological similarities between NHPs and humans make comparisons more relevant than with other species.

The cortical evoked potentials elicited by electrical stimulation of the soft ABI provide insights that are closer to conscious perception than those obtained from brainstem potentials. Today, clinicians depend on eABRs to guide accurate ABI placement during surgery and to assess the efficacy of central auditory prosthetics electrophysiologically. By contrast, eAEP recordings have several advantages: they enable the observation of the response of the highest level of the central auditory pathway, which is much closer to actual perception, and they may also more accurately reflect the tonotopic organization of the central auditory pathway. Indeed, eABR thresholds, as measured intra-operatively, were found to be lower than those defined from cortical responses. This disparity suggests that lower stimulation currents might be sufficient to generate eABRs, yet they may not guarantee that the signal reaches the cortex. Moreover, the use of a multichannel array enables access to cortical tonotopy, thereby allowing the fine-tuning of stimulation parameters through direct comparison with acoustic responses at the level of signal integration.

The NHP model enabled the monitoring of the stability of the soft ABI over a period of 4 months. Despite the electrodes maintaining relatively stable electrochemical properties (Supplementary Fig. 10a) and the AEP amplitudes remaining consistent (Supplementary Fig. 10b), we observed, both at the electrophysiological and behavioural level, an increase of activation thresholds over time. ABI migration has been observed in human cases, and CT and MRI imaging do not have the resolution to rule out a minor degree of migration of the soft ABI (primarily due to the small device size). Factors such as micromotion of the brainstem, or a foreign body reaction could contribute to increased thresholds, even while the capability of recording AECNRs remains intact (Supplementary Fig. 8b). However, we did not measure the evolution of electrode impedances over time.

Finally, our behavioural studies indicate that electrophysiological responses to electrical stimulation play a role in decision making. The fully electrical eTask is particularly relevant for comparing the responses of NHPs to those of human ABI users. One essential difference between our NHP subjects and human ABI users is that our NHP model has intact acoustic hearing. By contrast, ABI recipients have profound hearing loss in the implanted ear due to cochlear nerve damage or sectioning during tumour removal. In our bTask, we combined ABI electrical stimulation with acoustic stimulation^25^. The outcomes of this task produced a perceptual shift, showing that the animals responded to both types of stimuli (acoustic and electrical) as if they were of similar nature. This finding is important as it demonstrates the integration of different sensory inputs.

It is well established in the primate research community that comparing performances across behavioural tasks is a challenge, especially when considering complex tasks with daily motivational and attentional variability. However, the global performance of our NHPs in the tasks was consistent with comparable studies^31^. We found that our subjects could discriminate acoustic frequencies down to a 2 Hz difference, with reference frequencies ranging from 800 Hz to 10 kHz, which is slightly better than reported values for NHPs^32–37^. However, the specificity of the task as well as the intensive and long-lasting training could explain such high performance. Although the auditory task took over a year to reach asymptotic performance, later introduction of electrical stimuli elicited behavioural responses with little further training. This result suggests that the NHP was interpreting electrical stimulus as a sound, similar to humans implanted with ABIs and cochlear implants. Of course, with longer training periods (months to years), humans, and perhaps the animal, increase their performance via implants for complex signals such as speech.

In addition, electrodes 7 to 11 did not elicit a robust cortical response when stimulated below 1.1 mA in a single-pulse paradigm (Fig. 3c). However, these electrodes were used in a bipolar configuration during the behavioural experiments to better confine the electric field (Fig. 4i). Moreover, they were able to record AECNR when stimulated acoustically (Supplementary Fig. 8a,b). Combined with histological sectioning (Fig. 2g), these findings suggest that these electrodes may have been positioned near the surface of a region containing deep auditory neurons, which are harder to activate with electrical stimulation. However, we cannot completely rule out the possibility that these electrodes provided non-auditory sensation to the animal.

The surgical procedure and ABI implantation were performed uneventfully in our first subject, resulting in the collection of electrophysiology and behavioural data over several months. However, the procedure for the second subject was associated with complications including a displacement of the ABI (observed intra-operatively) and a prolonged recovery period. Consequently, this animal could not be subjected to behavioural testing with electrical stimulation. However, in response to auditory stimuli, this subject confidently performed tasks and had clear electrophysiological responses. The challenges encountered with this second subject underscore the complexity inherent in ABI surgery, especially in NHPs. Despite these difficulties, the extensive number of behavioural sessions conducted with our first subject, along with the range of parameters and control conditions tested, provides substantial insights into the specificity and resolution capabilities of the soft ABI. This comprehensive approach contributes valuable data towards the ongoing development and refinement of ABI technology.

In this study, we implemented a soft neurotechnology prosthesis onto the central nervous system to produce specific auditory percepts in an NHP model. The efficacy of the implant stimulation was assessed through intra-operative, electrophysiological and behavioural characterization, all of which led to a comprehensive benchmark for auditory neuroprostheses. The results support the concept that a soft multichannel ABI can efficiently stimulate the cochlear nucleus and produce high-resolution auditory percepts without secondary effects. The soft, miniaturized implants, together with the NHP model, offer a unique opportunity to develop and optimize central auditory neuroprostheses.

## Outlook

Further advances are needed before the clinical translation of soft brainstem electrode arrays for individuals with hearing loss who are not candidates for cochlear implants.

The introduction of microfabrication and soft polymer carriers in the manufacturing of the ABI represents a considerable advance over traditional neural implants. These innovations allow for implants with a higher electrode count, smaller electrode sites and improved conformability to contact the cochlear nucleus. By contrast, the current clinical ABI, relying on stiff, macroscopic components and highly manual manufacturing processes, is limited in electrode densification and tissue conformability.

The approach presented here can be further refined to increase the electrode density and associated selectivity of the implant using multilayered techniques and advanced lithography techniques, all without compromising mechanical compliance. This approach would facilitate ultradense electrode arrays, enabling a broader range of spatiotemporal stimulation patterns and in situ programming, thereby providing clinicians with greater versatility in personalized modulation. It is also important to anticipate the subsequent integration and interface of the soft microfabricated implants with the electronic hardware of clinical receiver and stimulator electronics^38^.

Second, conformability often results in implants that may be delicate to manipulate and surgically handle. In this study, we proposed and tested several options to improve the robustness and handling of soft miniaturized implants. A stiff resorbable spine was essential for the grasping and insertion of the soft ABI.

Third, complete encapsulation of metallic interconnects within polyimide films is an important design step towards hermetic and long-term functionality of the implants in vivo. Further development calls for flexible, thin-film permeation barrier technology. In long-term implantation scenarios, the failure mechanisms of macroscopic clinical devices and thin film-based devices really differ owing to their structural characteristics. Macroscopic devices, composed of bulk metallic wires coated with insulated fluorinated polymers then embedded in polymeric tubing and metallic discs, fail primarily owing to mechanical damage from cyclic movements or interchannel breakdown. The physical separation between individual cables or electrodes, typically ranging from hundreds of microns to millimetres, limits cross-channel shorts. In addition, the substantial metal volumes can mitigate the impact of partial surface corrosion. By contrast, thin film devices are more vulnerable owing to their reduced dimensions. The close proximity of layers and tracks, often only tens of micrometres apart, increases susceptibility to interchannel crosstalk following water ingress. This risk of device failure is further compounded by the rapid corrosion of the thin metallic layers. While the potential delamination of the different layers is a critical issue to consider, mere improvements in adhesion are insufficient to ensure device functionality in the long term. Even as water and ions permeate through perfectly adherent polymeric layers, it can still lead to crosstalk and metal corrosion. Therefore, hermetic barrier layers are crucial for the long-term reliability of thin film devices^38,39^. Meticulous and often iterative technological advancements are essential in translational research and imperative to bring a new-generation ABI and other implantable electrodes to clinical reality.

These technological features along with more general auditory responses, can be efficiently tested and analysed using the auditory cortex readout provided by this novel NHP model.

## Methods

### Microfabrication

The process detailed below depicts the fabrication process for both soft ABI and ECoG devices used in this study (design and dimensions in Supplementary Fig. 1).

The fabrication of the micropatterned interconnects (Supplementary Fig. 2a) began with the evaporation (EVA 760, Alliance-Concept) of a titanium film (Ti, 50 nm), followed by aluminum (Al, 200 nm) on a 4-inch silicon wafer that was previously dehydrated (UM100, Memmert) and treated with oxygen plasma. Next, the wafer surface was once again dehydrated at 150 °C for 15 min (UM100, Memmert) and cleaned from organic contaminants with an oxygen plasma treatment (GiGAbatch, Tepla at 200 W, 0.8 mbar, 400 sccm, 2 min). Soon after, a 25-nm layer of SiO_x_ and a 5-nm layer of Ti were deposited by sputtering (SPIDER600, Pfeiffer). Following another dehydration step in a convection oven and O_2_ plasma activation (600 W, 0.8 mbar, 400 sccm, 1 min), the wafer surface was covered for 30 s with a 0.1% solution of APTES in isopropanol (VM652, HD MicroSystems) before being spin dried. The wafer was then coated with the first layer of polyimide (1 µm PI2610, HD MicroSystems). The curing of the photoresist was performed in three stages: (1) soft bake at 70 °C for 3 min, (2) soft bake at 110 °C for 3 min, and (3) a hard bake at 200 °C under nitrogen atmosphere in a convection oven (Heraeus T6060).

Next, the wafer surface was dehydrated (150 °C, 15 min) and plasma activated (200 W, 0.5 mbar, 200 sccm O_2_, 30 s), after which the Ti/Pt/Ti layers (25/100/25 nm) were deposited via sputtering. The patterning of the metal films then began with the spin coating of a 0.7-µm layer of photoresist (AZ 10XT-07, MicroChemicals). The resist was baked at 110 °C for 90 s before being exposed using a mask aligner (MA6 gen3, Süss). The illumination dose was set to 170 mJ cm^−2^ and the Cr mask, previously patterned via direct-write lithography, was placed in hard contact with the wafer. The wafer was then processed in a modular cluster tool (ACS 200, Süss) where the photoresist was hard baked (110 °C, 90 s) and developed for 87 s using a KOH-based solution (AZ 400 K, MicroChemicals). Next, a reflow of the photoresist was performed by placing the wafer on a hotplate at 120 °C for 2 min. This treatment was intended to round off the patterns profile to mitigate the redeposition of material on the photoresist sidewalls during the subsequent removal of the Ti/Pt/Ti films via ion beam etching (IBE) (Nexus IBE350, Veeco). During the etching procedure, the wafer was tilted at a 10° angle so as to further reduce the buildup of metallic fences. The remaining photoresist was stripped using a combination of O_2_ plasma (200 W, 0.5 mbar, 200 sccm O_2_, 30 s) and solvent baths (Microposit Remover 1165, Dow Chemicals) at 70 °C.

Following the complete removal of the photoresist, the wafer surface was dehydrated (150 °C, 15 min) and plasma activated (200 W, 0.5 mbar, 200 sccm O_2_, 30 s) before going through another round of silanization (VM652, HD MicroSystems). Next, the wafer was coated with the second layer of polyimide (1 µm PI2610, HD MicroSystems). The curing of the polyimide was performed as described above for the first layer. Following another dehydration step (150 °C, 15 min) and oxygen plasma (200 W, 0.5 mbar, 200 sccm O_2_, 30 s), the Ti/SiO_x_ films (5/25 nm) were deposited via sputtering (SPIDER600, Pfeiffer). The patterning of the shell layers began with the coating of a 4-µm-thick layer of photoresist (AZ ECI3027, MicroChemicals). Proper adhesion between the SiO_x_ and the resist was ensured with hexamethyldisilazane (HMDS) treatment before spin coating. The photoresist was exposed using a mask aligner (MA6 gen3, Süss, 560 mJ cm^−2^, hard contact). This alignment procedure is critical to the success of the process: a misalignment of less than 1 µm is required to ensure the proper encapsulation of the core layers. The exposed photoresist layer was developed in an automatic cluster tool (MF CD 26, Shipley Microposit, 137 s). Next, the SiO_x_/Ti/PI/Ti/SiO_x_ layers were dry etched using a sequence of CHF_3_/He, O_2_ and CHF_3_/He chemistries (Advanced Plasma System, SPTS). Upon completion of the etching procedure, the remaining photoresist was stripped using a combination of O_2_ plasma (200 W, 0.5 mbar, 200 sccm O_2_, 30 s) and sonication in a solvent bath (Microposit Remover 1165, Dow Chemicals) at 70 °C.

The opening of the electrodes and contact pads was then performed in a third lithographic step. First, the wafer was dehydrated (125 °C, 3 min) and coated with a 4-μm-thick layer of photoresist (AZ ECI3027, MicroChemicals). Next, the photoresist was exposed and developed using the same procedure as before, albeit with a slightly higher exposure dose (600 mJ cm^−2^). Once the electrodes and contacts were exposed, the top SiO_x_/Ti and polyimide layers were then etched using the same chemistries as before. To prevent surface oxidation of the exposed core layers, the top Ti of the Ti/Pt/Ti stack was also removed at this stage via IBE (10° tilt). Finally, the photoresist mask was stripped following the sequence of O_2_ plasma (200 W, 0.5 mbar, 200 sccm O_2_, 30 s) and solvent baths (Microposit Remover 1165, Dow Chemicals) at 70 °C.

The fabrication of the silicone encapsulation (Supplementary Fig. 2b) started with the manufacture of a carrier for the top polydimethysiloxane (PDMS) membrane. The process began by adding the silicone monomer (Sylgard 184 part A, Dow Corning) to the cross-linker (Sylgard 184 part B, Dow Corning) using a 10:1 weight ratio between the two. The blend was mixed and degassed (ARE-250, Thinky) before being spin coated (EL S 200, Obducat) on a 4-inch silicon wafer at 3,200 r.p.m. Following a 10-min reflow period on a flat surface, the PDMS layer was cured in a convection oven (GP/40/F/SS/250 ELITE) at 75 °C for 2 h. Next, manufacture of the top encapsulation itself began. A first polyethylene terephthalate (PET) sheet of 23 µm (DuPont Mylar 23 A, Lohmann Technologies) was manually laminated onto the carrier wafer and cut to shape with a razor blade before being wiped with isopropanol (IPA). A new batch of PDMS was prepared following the same process as before and a 75-µm-thick layer was spin coated directly onto the PET. Just as before, the fresh PDMS layer was cured at 75 °C for 2 h after a 10-min reflow. Afterwards, a second 23-µm-thick PET sheet was laminated and cut to shape with a razor blade. The PET/PDMS/PET stack was then peeled off the carrier (Si/PDMS), which could then be reused for the creation of additional encapsulation stacks.

Next, femto-laser machining (WS-TURRET 200, Optec) was used to cut the openings for the contacts and electrode sites in the fabricated encapsulation stack. To be bonded on top of the wafer containing the micropatterned interconnects, the PET/PDMS/PET stack was first mounted onto an acrylic disc using a thick PDMS slab as a weak adhesive between the two. The top PET sheet was then carefully peeled off, revealing the PDMS layer underneath. Both the interconnects wafer and the mounted encapsulation stack were then placed in a plasma oven (PCCE Nano, Diener) where an O_2_ treatment was performed (100 W, 0.2 mbar, 30 s) to activate the SiO_x_ and PDMS layers, respectively. The Si wafer and the acrylic disc were then mounted onto a custom alignment tool consisting of a vacuum holder attached to a *z*-axis micromanipulator atop of an *x*-*y*-*θ* stage. Using the pair of overhanging CCD cameras, the PET/PDMS encapsulation was then aligned with the structures present on the interconnects wafer before being lowered and pressed against it. Next, the stack was unloaded from the aligner, allowing for the acrylic disc and PDMS slab to be removed. The top PET film was kept in place, as it would later be used as a screen-printing mask. To complete the bonding process of the top PDMS membrane, the assembled stack was placed under a weight of 2 kg and kept in an oven at 75 °C for 12 h.

Following the top bonding sequence, the wafer surface was divided into multiple parts by laser machining (WS-TURRET 200, Optec) the PET/PDMS layer. This step was performed to facilitate the assembly of the bottom PDMS membrane. Next, the individual pieces of the PET/PDMS/PI/Pt/PI stack were released from the carrier Si wafer via anodic dissolution of the Al layer. To do so, the wafer was placed in a sodium chloride solution (NaCl in H_2_O, 15 g l^−1^) alongside a Pt counter electrode, and a potential of 1.5 V was applied between the two for ~8 h (refs. ^40,41^). The released samples were washed in deionized water and dried at room temperature.

Next, manufacture of the bottom PDMS membrane began. Following an oxygen plasma treatment (200 W, 0.2 mbar, 5 min), a 4-inch silicon wafer was covered with a sacrificial layer of poly(sodium 4-styrenesulfonate) (PSS, Sigma Aldrich) by spin coating the solution at 3,200 r.p.m. A 50-µm layer of PDMS (Sylgard 184, Dow Corning, 10:1 ratio) was then spin coated on top and cured in an oven at 75 °C for 2 h. Next, a second PDMS layer of 20 µm was spin coated on top of the first one, but this time the curing process was interrupted as soon as the gel point was reached (approximately after 15 min at 75 °C). Immediately after, the wafer and the pieces of the PET/PDMS/PI/Pt/PI stack were placed in a plasma oven (PCCE Nano, Diener) where an O_2_ treatment was performed (100 W, 0.2 mbar, 30 s) to activate the SiO_x_ and PDMS. The bottom bonding sequence was performed manually by carefully laying down the individual stack parts on the PDMS, with the PET side facing up. Finally, the curing of the PDMS was completed by placing the wafer back in an oven at 75 °C for 2 h.

Electrode coating and connector assembly (Supplementary Fig. 2c) began with the preparation of the Pt-PDMS composite for the coating of the electrode sites^21^. First, a fresh batch of PDMS (Sylgard 184, Dow Corning) was prepared using a 2:1 weight ratio between the monomer and the cross-linker. Next, an organic solvent (cyclohexane, Sigma Aldrich) was added to obtain a 2:1 cyclohexane:PDMS blend. Of the obtained solution, 742 µl were then pipetted in a glass Petri dish and mixed with 500 mg of Pt particles (diameter 0.27–0.47 µm, Strem Chemicals) using a spatula. Once the mixture was homogeneous, the cyclohexane was evaporated under vacuum, resulting in a conductive composite with a PDMS:Pt ratio of 30% wt and a working time of 2–3 h. This procedure was followed for the composite of the implants for monkey L. For monkey G, the composite was provided by Neurosoft Bioelectronics.

Next, the surface of the wafer prepared previously was activated via an O_2_ plasma treatment (Diener Zepto, 20 W, 0.2 mbar, 30 s). Immediately after, the Pt-PDMS composite was screen printed onto the electrode sites and contact pads, using the top PET film as a stencil mask. Once this was done, the PET layer was carefully peeled off, exposing the top PDMS encapsulation.

Following the removal of the PET, the connector, in the form of a flexible PCB (FPCB), was placed on top of the contact using a custom pick-and-place platform. The FPCB was custom designed in Autodesk Eagle and manufactured by a third party. Finer details of the design, which exceed standard manufacturing capabilities, were realized afterwards via femto-laser machining. Following proper placement, the connector was secured using silicone (RTV 734, Dow Corning). The whole assembly was left to rest overnight at room temperature before the final curing step in a convection oven (Binder Model BD 23, 55 °C, 3 h). The outline of the individual devices was then defined by laser cutting through both the top and bottom silicone encapsulation. The resulting structures were released from the carrier wafer by dissolving the sacrificial PSS layer in a bath of deionized water.

### Pedestal system fabrication and assembly

The system is composed of a transdermal port named ‘pedestal’, embedding the connector mounted onto a footplate that is fixed onto the skull of the animal (Supplementary Fig. 5). The implants were interfaced with two independent ‘pedestals’ (one for the ABI and one for the ECoG) that were fixed onto the footplate in two distinct surgeries (Supplementary Fig. 5c,d). The connector of each implant was then available independently (Supplementary Fig. 5e).

The pedestal, footplate and inserts were machined using traditional computer numerical control (CNC) manufacturing techniques in the mechanical workshop of the Ecole Polytechnique Fédérale de Lausanne (ATME EPFL, Lausanne, Switzerland) in poly-ether-ether-ketone (PEEK) base material (PEEK-CLASSIX LSG 1, Angst+Pfister). All the received pieces were cleaned in acetone and isopropanol before use to remove machine oil. Into the inserts, M1 stainless steel screws (BN 402, Bossard) were screwed for adherence to the magnets in the cap. These could be removed for MRI acquisitions. The protective caps were 3D printed using stereolithography (Formlabs 3 using Grey Resin, Formlabs) in which the magnets were glued into the protective caps using standard cyanoacrylate instant glue (Loctite 4242, Loctite). The magnets are neodymium discs of 2-mm diameter and 1-mm thickness (S-02-01-N, Supermagnete). The inserts were glued into the PEEK pedestals and subsequently, the connector mounted onto the implant was glued (using cyanoacrylate glue) in place as well. The connectors are two Omnetics connectors with 18 and 36 pins, respectively (Omnetics). Next, the backside of the pedestal was filled up with silicone sealant (RTV 734, Dow Corning) until the rim of the pedestal was filled, leaving no voids and allowing the FlexComb connector^22^ and ground platinum wire to exit the pedestal from the side. The pedestal was screwed onto the footplate using custom-made M1.6 titanium screws with the same header as clinical titanium self-drilling bone screws that were used to fix the footplate onto the skull (TiMesh M1.6 × 3.5 mm self-drilling, Medtronic). Before surgeries, all devices (soft ABI and soft ECoG) and the pedestal system were sterilized using hydrogen peroxide gas plasma.

### Electrode characterization

In vitro EIS was performed using a potentiostat (Reference 600, Gamry Instruments or Autolab PGSTAT128N, Metrohm) in a 3-electrode configuration. The Ag/AgCl reference electrode, platinum counter electrode and implant under test (working electrode) were immersed in phosphate buffered saline (PBS) (1×), and the impedance spectrum was measured between 0.1 Hz and 1 MHz (or 500 kHz, in the case of the Autolab) using an input signal amplitude of 100 mV (root-mean-square).

Voltage transients were measured in PBS (1×) upon the application of symmetric, biphasic, cathodic-first, current pulses between the electrode under test and a platinum counter electrode. The pulses amplitude was set to 100 µA and their duration to 600 µs (300 µs per phase), with an interpulse period of 10 ms. The resulting VTs were collected via an oscilloscope (MDO3014, Tektronix) connected across the two poles of the current pulse generator (ISP2100, A-M Systems). For each device, the access voltage, that is, the purely resistive drop occurring at the onset of a current pulse, was estimated by averaging the voltage values over the first µs of each phase. In vivo VTs were collected using the same setup but this time with the counter electrode connected with the ground wire (perfluoroalkoxy (PFA)-coated platinum wire (127 µm bare diameter); A-M Systems, 773000) with exposed tip. A breakout PCB connected inside the pedestal allowed easy access to the individual channels.

The maximum charge injection capacity was measured in PBS (1×) using a platinum electrode as counter and an Ag/AgCl electrode as reference, by looking at the interface polarization at increasing currents. The maximum was defined by the current for which the interface polarization was larger than the water window (−0.6 V, defined for plain platinum), following the guidelines from ref. ^23^.

### Animal model

All procedures were done in agreement with the Swiss regulation for animal experimentation, under License number 2020-06E-FR, and performed at the Swiss Non-Human Primate Competence Center for Research. Two female rhesus macaques (*Macaca mulatta*, monkey L and monkey G), 6 and 9 years old, were used in this study. Preliminary terminal acute experiments were also conducted at the Washington National Primate Research Center at the University of Seattle (through the Tissue Donation Program) for understanding ABI design constraints and in view of validating the experimental approach.

### Anaesthesia for ECoG and ABI implantation

Surgical procedures were performed in agreement with the Swiss regulation mentioned above under deep sedation. Induction was done using a mixture of ketasol (10 mg kg^−1^) and midazolam (0.3 mg kg^−1^), and followed by an intravenous anaesthesia of 1% propofol (10 mg kg^−1^) and remifentanil (0.025 mg ml^−1^) throughout the entire procedure. Propofol bolus was punctually administered when needed (vital constant check). This anaesthesia protocol affects brain activity and recordings. ABR recordings made across surgical procedures as a control for brain activity had a substantial decrease in basal cortical signal. This may have affected in particular eABR recordings that were recorded after 7 h of intense surgery.

### EcoG implantation over the right auditory cortex

After anaesthesia, the head of the animal was shaved and cleaned before bringing it to the surgical table. The animal was intubated to guarantee proper oxygenation, and all relevant vital parameters were monitored throughout the procedure. Once placed in the stereotactic frame, the animal was draped and cleaned following proper sterile techniques. The skin was opened in an L shape on the right side. Muscles were retracted to clear the skull and allow visualization of bony landmarks. A cranial window was made around −8 to −18 mm from bregma and 1.89 cm above the right ear (Supplementary Fig. 6a,b). The dura was then opened in an L shape, and after identification of the auditory cortex, the ECoG was placed subdurally (Supplementary Fig. 6c). The implant was then secured at the entrance of the craniotomy by screwing the custom-made wings, and the pedestal was then screwed to the skull as well (Supplementary Fig. 6d). The dura mater was then sutured and a piece of muscle was placed around the opening of the dura mater with the flex cable of the implant to prevent CSF leakage. The ECoG platinum ground wire (PFA-coated platinum wire (127 µm bare diameter); A-M Systems, 773000) with exposed tip was placed epidurally and below the skull in the frontal part of the craniotomy. The bone flap was placed back and secured using titanium bridges (Supplementary Fig. 6d). Dental cement (PalacosR+G, Heraeus) was applied on the bone to protect the flex cable of the ECoG from the pedestal to the craniotomy entry. The skin was then closed and sutured tight around the pedestal. Post-operative care was administered and the animal was left to recover for ~1 week.

### Soft ABI implantation onto the left cochlear nucleus

After anaesthesia, the head of the animal was shaved and cleaned. The animal was intubated and placed in a right lateral decubitus position. The skin was incised, and the sternocleidomastoid muscle and nuchal muscles were retracted to allow visualization of the mastoid surface and the suboccipital region. Under 3D exoscopic view (Storz), a small osteoclastic craniotomy was drilled, the sigmoid and transverse sinus decompressed, and the dura mater exposed. After incision of the dura and fixation of the dural flaps, the cisterna magna was opened, allowing deliquorization to gain surgical exposure. The cerebellum was then protected by a neuropatty and gently pushed posteriorly to allow cranial nerve dissection and identification (Supplementary Fig. 3a). Under endoscopic view (Storz), the soft ABI array was placed in the lateral recess of the fourth ventricle using a curved cup-forceps and secured using a muscle patch and fibrin glue (TISSEEL, 4 ml, Baxter) (Supplementary Fig. 3c). Ground platinum wire (PFA-coated platinum wire (127 µm bare diameter); A-M Systems, 773000) with exposed tip (used as a return electrode for monopolar stimulation) was placed under the muscle near the cranial window. A watertight closure of the dura was achieved by the placement of artificial dura, additional muscle patch and fibrin glue. The dissected muscles were repositioned, and the skin was closed and sutured tightly around the pedestal. The animal was left to recover for 2 weeks with appropriate post-operative care.

### ABR/eABR recordings and analysis

Auditory brainstem responses (ABRs, eABRs) were recorded under sedation, using the Medtronic NIM-ECLIPSE IONM System and corresponding ABR program. Two recording needle electrodes were placed subcutaneously at the base of the mastoid bone below each ear, one reference electrode was placed at at the vertex of the cranium, Cz (midline central), and one ground electrode under the skin at the back of the neck or at the eyebrow level (Fig. 2a). For ABRs, clicks were presented through speakers placed directly in each ear at 21 clicks s^−1^ for 512 (or 1,024) repetitions at increasing sound level (40–120 dB SPL). For eABRs, electric stimuli presented to the soft ABI were pulses (from 0.01 to 0.7 mA amplitude, 300 µs duration) at 13 pulses s^−1^.

ABR and eABR signals were filtered (300–3,000 Hz for ABR, 10–4,000 Hz for eABR) and averaged (using all repetitions), all online for direct visualization. For eABRs, the first 1 ms of the response was set to zero to hide the biggest contribution of the stimulation artefact (can be seen in Supplementary Fig. 4).

### AEP/eAEP recordings and analysis

AEP and eAEP were recorded in awake and in sedated conditions. For AEP awake recordings, tones were controlled and generated using the Synapse software of TDT (RZ6/RZ2), through SA1 stereo power amplifier and MF1 multifield magnetic speaker, while the animal was sitting in a modified double-walled electrically shielded sound-proof room (compact model, type AB200, Eckel Industries), with the speaker at ~30 cm from the animal’s head. For AEP sedated recordings, tones were generated either using the same setup with speakers at ~5 cm, or using custom MATLAB script and Etymotic speakers placed directly in the animal’s ears. AEP stimulus was a sequence of pure tones (500 ms on, 500 ms off) at different frequencies (from 300 Hz to 10 kHz) and different sound levels (calibrated using a MiniDSP UMIK microphone and the REW software, with A weighting). eAEPs electric stimuli presented to the soft ABI were pulses with 0.05–2 mA amplitude of 100–600 µs duration delivered at 1 or 1.25 pulses s^−1^ using a Medtronic NIM-ECLIPSE IONM System. Signals from the ECoG were recorded using the W2100-HS32 wireless recording system from Multi Channels Systems, in parallel to a tone or pulse synchronization signal, for alignment of the recorded data with stimulus onset. Signals were then post processed using custom MATLAB scripts, by filtering (generally 50 Hz Notch and 1–100 Hz bandpass using 2nd-order Butterworth filter (‘butter’ and ‘filtfilt’ functions from MATLAB)), followed by synchronization and averaging (AEP over ~120 repetitions, eAEP over ~30 repetitions). When needed for properly comparing channels and AEP amplitudes, the data were sometimes normalized using the MATLAB ‘zscore’ function. AEP amplitudes were measured by taking the maximum–minimum of the signal within a response window starting from 10 ms to 100 ms after sound onset. eAEP amplitudes were measured by taking the maximum–minimum of the signal within a response window starting from 20 ms (to avoid electrical artefact) to 250 ms after stimulation onset. Correlation coefficients were calculated over the response window using the MATLAB ‘corr’ function. The random matrix (with the same sample size) was generated using the MATLAB ‘rand’ function. Comparisons were performed using two-sided *t*-tests.

### AECNR recordings and analysis

AECNRs were recorded and processed similarly to AEPs. AECNR amplitudes were taken as the difference between the maximum and minimum of the signal within a 10–100-ms window following tone onset.

### CT/MRI imaging

CT images were acquired with 670-µm-thick slices on a PHILIPS-705 scanner. MR images were taken on a GE Medical Systems Scanner 3 T using different sequences (T1, T2, TSE, BRAVO-isotropic hypersense). CT and MR image co-registration was performed manually using the ITK-snap software.

### Acoustic discrimination task setup

The animal was trained in a discrimination task^25^ to differentiate tones, using positive reinforcement with food rewards (no water restriction) while sitting in a primate chair with arms free inside a modified double-walled electrically shielded sound-proof room (compact model, type AB200, Eckel Industries) (Supplementary Fig. 9). Training lasted for more than 1 year. Auditory stimulation sequences included a series of reference and discrimination tones, both randomly chosen from a predefined set, over each trial. All trials were self-initiated by the animal by the initial press of the lever. The animal was then trained to release the lever (‘go now’) within the discrimination period when the presented discrimination tone was different from the reference tone, or keep the lever pressed (‘hold’) when the animal could not perceive a difference between the reference and discrimination tone (Fig. 4a). All tones were pure tones of various frequencies (ranging from 500 Hz–10 kHz with positive/negative frequency difference from 2 Hz to 2 kHz) of 100-ms duration with a 10 ms rise/fall time, played at 10 dBV through TDT SA1 stereo power amplifier and TDT MF1 multifield magnetic speakers. The automation of the task was done through TDT RZ2 Processor, which would collect the lever signal to trigger the start of the trial, record all task variables and control the delivery of the reward signal. All components of the task were controlled with a custom Synapse experiment using the Pynapse embedded Python editor.

### Electrical stimulation within the discrimination task

Electrical stimulation was introduced to the discrimination task to evaluate the animal’s performance in this modality. In the bTask (Fig. 4b, left), the acoustic tone and electrical pulse were synchronized to be delivered at the same time. The stimulator was triggered digitally, and the stimulation pulse was delivered using the Medtronic NIM-ECLIPSE IONM System to the soft ABI. Stimulation parameters varied from 0.1 to 2 mA amplitude, 150–600 µs pulse width and from single pulses to bursts at 50–100 pulses s^−1^ with a fixed 100-ms burst duration and from different electrodes. For the eTask (Fig. 4b, right), another programmable stimulator (Model 3800 MultiStim, A-M Systems) was triggered while respecting the auditory task timing, with 100-ms burst duration. Stimulation parameters were also varied within the range 1–1.5 mA, 300–600 µs pulse width and 50–500 pulses s^−1^ bursts from various electrode pairs. The animal was rewarded during the electrical trials on the basis of theoretically expected behaviour, allowing for learning and progress. For both kinds of task (bTask and eTask), individual trials including electrical stimulation were almost always interleaved in a random fashion with purely auditory trials to evaluate day to day performance changes at ‘known’ trials.

### Task performance analysis

Variables from the task as recorded through the TDT RZ2 processor were then imported into MATLAB/Python for further analysis using custom-made scripts. Only trials that lasted for at least the duration of the reference tones and the first discrimination tones were kept, as only these trials could lead to an informed decision by the animal. Only durations less than 5 s were kept, as for any trials above that, the animal was considered not focused on the task. As the lever release time is a proxy for the animal’s response to the presented stimulus, it was used to evaluate the differences between trial types. Discrimination curves were drawn from the response time at each frequency’s absolute difference presented. Distributions were plotted as empirical cumulative distribution functions using the iqplot package in Python. Confidence intervals (95%) were computed using 10,000 bootstrap replicates. To avoid the assumption that the response times belonged to a normal distribution, they were statistically compared using non-parametric Mann–Whitney unpaired two-tailed *t*-tests in GraphPad Prism.

### Histology

Brain tissue was collected after euthanasia, perfusion of the animal and 24 h post fixation (4% paraformaldehyde). The tissue was then prepared for cryosection: samples were plunged in a sucrose bath (15% and 30%) for preservation before immersion in isopentane for quick freezing. Sections were then cut at 40-µm thickness using a cryostat and imaged using a slide scanner microscope (Olympus Slides Scanner VS120). Soft ABI tip curvature was estimated from a ×40 image in Fiji: points were manually fitted using the multipoint tool, following the implant’s imaging marker in the region of electrode sites. Coordinates were then exported into MATLAB for circle fitting, where the circle’s radius was extracted.

### Statistical analysis

Confidence intervals were taken as 95%. Median values are represented. *P* values are represented as *P < 0.05, **P < 0.01, ***P < 0.001 and ****P < 0.0001.

### Reporting summary

Further information on research design is available in the Nature Portfolio Reporting Summary linked to this article.

## Supplementary information

Main Supplementary Information Supplementary Figs. 1–10 and captions.

Reporting Summary

Supplementary Data Source data Supplementary Figs. 7–10.

## Source data

Source Data Figs. 1–4 Tab 1. Source data for Fig. 1e,f. Tab 2. Source data for Fig. 2b–g. Tab 3. Source data for Fig. 3b–e,g,h. Tab 4. Source data for Fig. 4c–i.

## Data Availability

Source data for the main figures in this study as well as MRI/CT scan images are available in Zenodo at 10.5281/zenodo.14865857 (ref. ^42^). Source data are provided with this paper.

## Code availability

The custom code for controlling the behavioural task through the Synapse software of TDT is available in Zenodo at 10.5281/zenodo.14865857 (ref. ^42^).
