# Supplementary material for: High-resolution prosthetic hearing with a soft auditory brainstem implant in macaques
Source: Nat Biomed Eng. 2025 Apr 18;9(9):1403–17. doi: 10.1038/s41551-025-01378-9 (PMC12443620; doi:10.1038/s41551-025-01378-9)
Supplement: Supplementary file 1 — Supplementary Figs. 1–10 and captions. [file 41551_2025_1378_MOESM1_ESM.pdf]

nature biomedical engineering

Supplementary information https://doi.org/10.1038/s41551-025-01378-9

High-resolution prosthetic hearing with a soft auditory brainstem implant in macaques

In the format provided by the authors and unedited

SI1

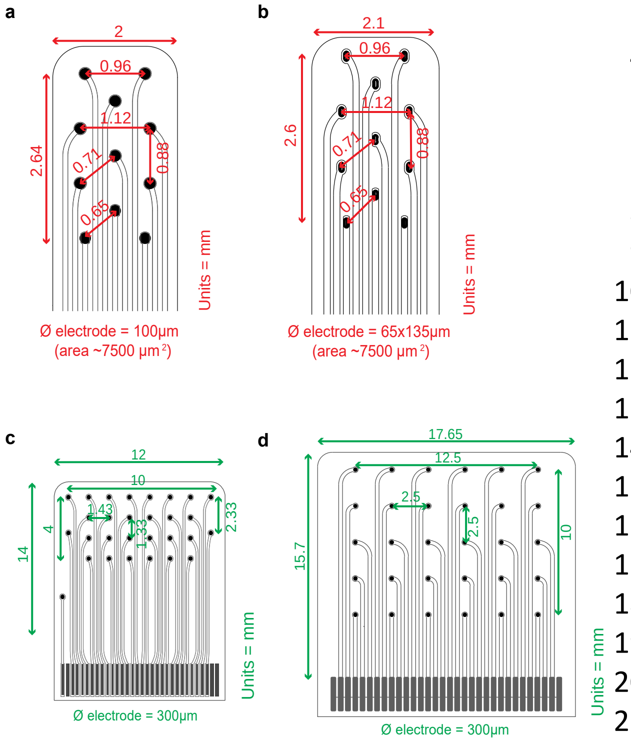

Supplementary Fig.1 | Soft ABI and ECoG designs showing distances between electrodes and tracks. a. Soft ABI design and dimensions. First iteration used for monkey L. **b.** Soft ABI design and dimensions. Second iteration used for monkey G. **c.** ECoG design and dimensions. First iteration used for monkey L. **d.** ECoG design and dimensions. Second iteration used for monkey G.

SI2

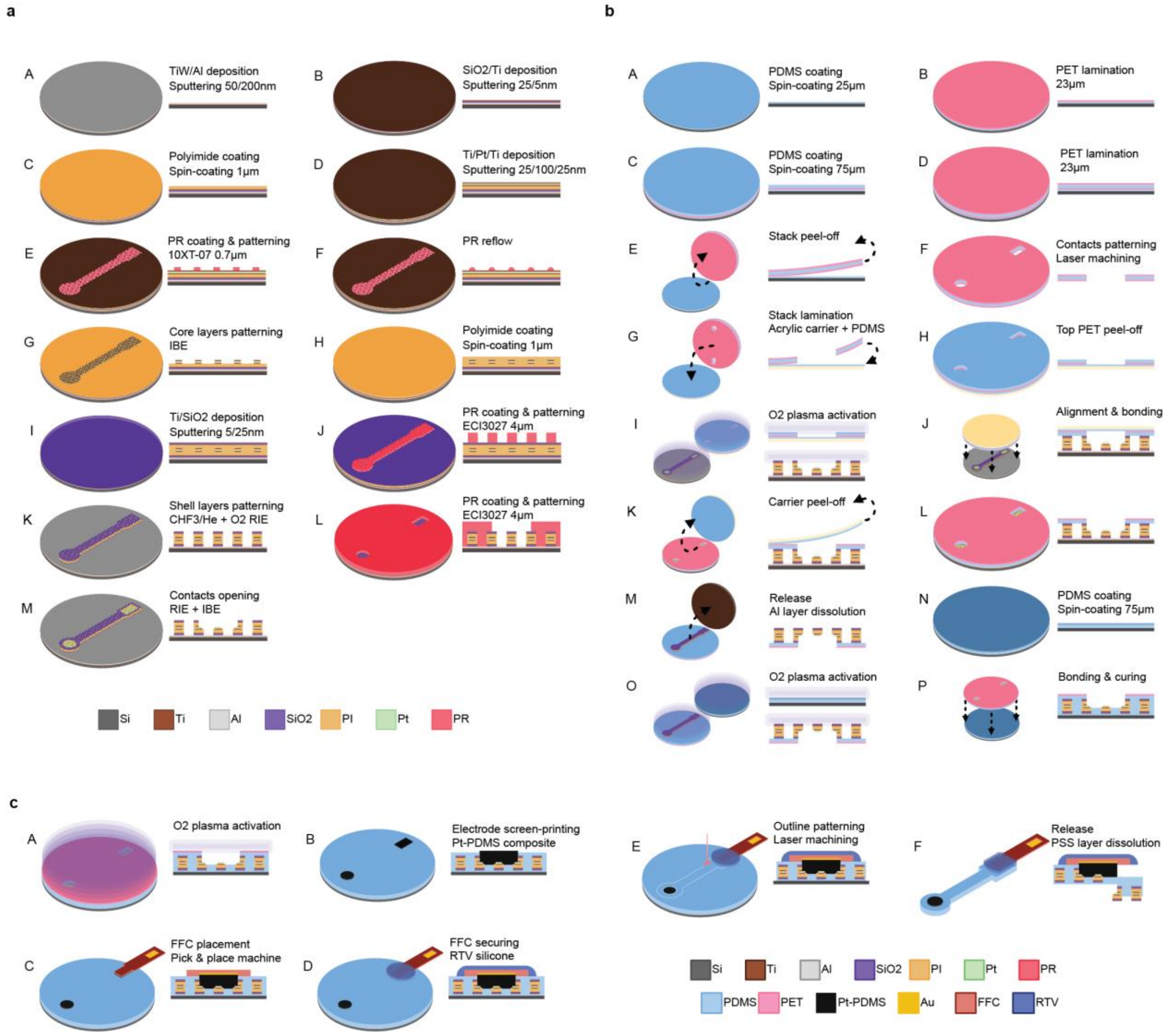

Supplementary Fig. 2 | Devices microfabrication steps. a. Micropatterned interconnects. **b.** Silicone encapsulation. **c.** Electrode coating and connector assembly.

SI3

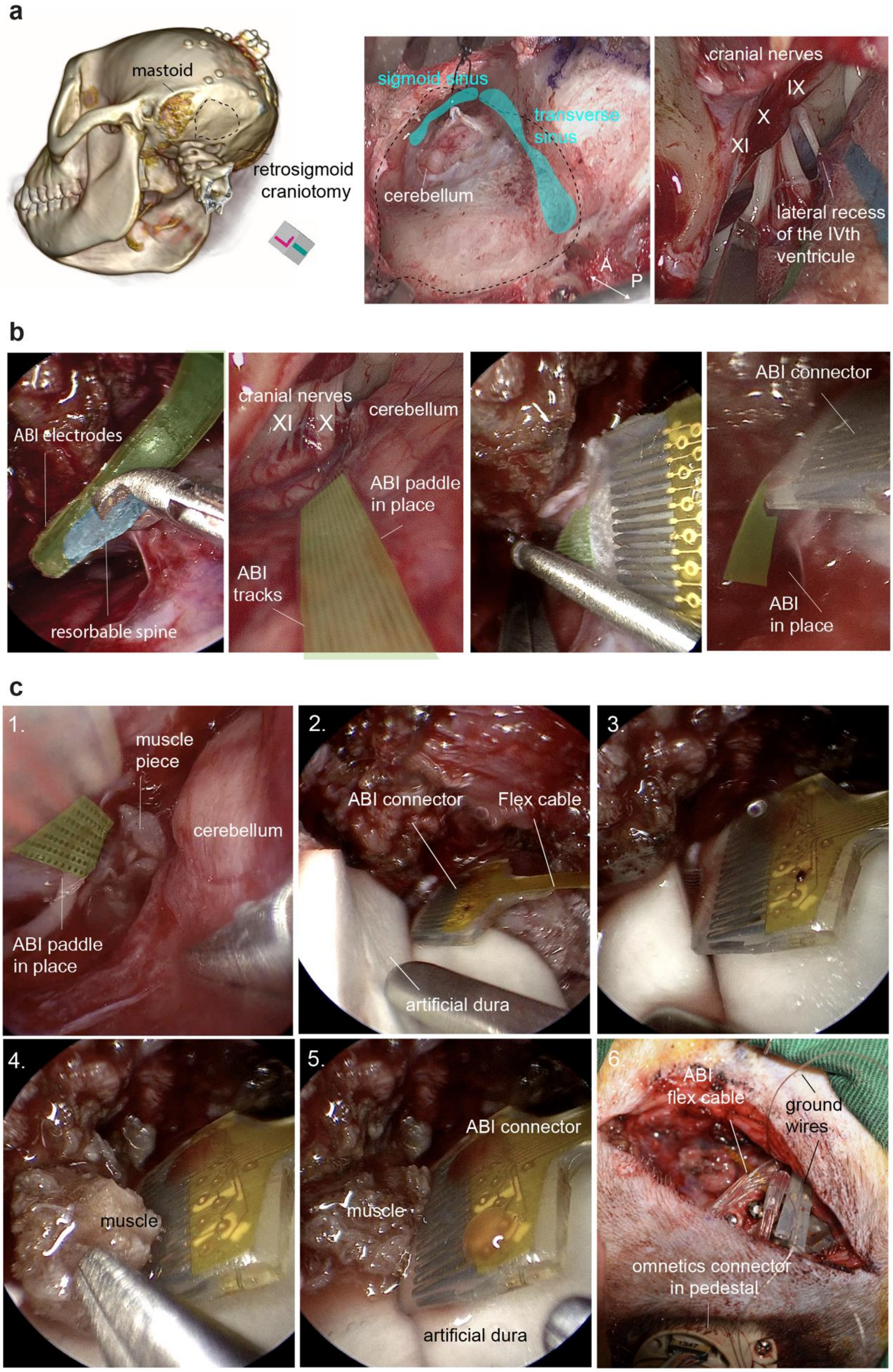
a
mastoid
retrosigmoid
craniotomy
sigmoid sinus
transverse
sinus
cerebellum
A
P
cranial nerves
IX
X
XI
lateral recess
of the IVth
ventricule
b
ABI electrodes
resorbable spine
cranial nerves
XI
X
cerebellum
ABI paddle
in place
ABI
tracks
ABI connector
ABI
in place
c
1.
muscle
piece
cerebellum
ABI paddle
in place
2.
ABI connector
Flex cable
artificial dura
3.
4.
muscle
5.
ABI connector
muscle
artificial dura
6.
ABI
flex cable
ground
wires
omnetics connector
in pedestal

Supplementary Fig. 3 | Soft ABI implantation surgical procedure. a. Surgical approach and cranial nerves dissection (see Methods for details). The dura was opened below the sigmoid and transverse sinuses (colored in blue on the picture), and the cerebellum was pushed on the side to allow cranial nerves visualization. These structures serve as landmarks to place the soft ABI on the CN. **b.** ABI handling and placement. Special-purpose forceps were used to grab the resorbable spine (colored in light blue), allowing for the soft ABI (colored in light green) to be inserted in the lateral recess of the IVth ventricle. Following electrophysiological tests, the cerebellum was pushed back in place. **c.** ABI securing. A muscle piece is grafted to fill the space between the cerebellum and the dura. Artificial dura was affixed around the connector to help secure the ABI array and connector in place. Finally, fibrin sealed the durotomy just below the FlexComb connector that exits the craniotomy. The flex cable was screwed on the skull further up using the anchoring wings.

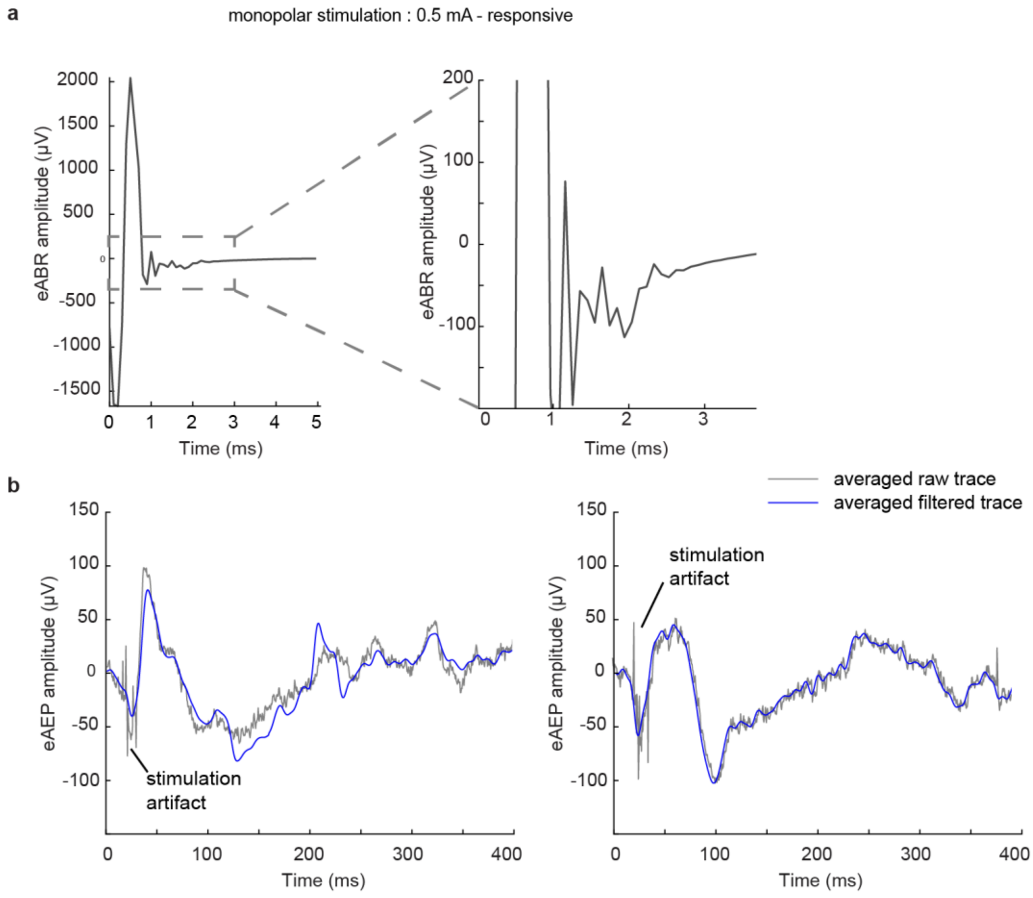

Supplementary Fig. 4 | eABR and eAEP measurements and artifacts. a. Examples of intraoperative eABR traces considered as responsive, with a focus on the portion of the signal following the large artifact. **b.** Two examples of eAEP traces in response to soft ABI stimulation. The gray trace is the raw data averaged over the stimulation period and displaying the stimulation artifact prior to the response. The blue trace displays the same data but filtered (notch at 50 Hz and bandpass 1 - 100 Hz).

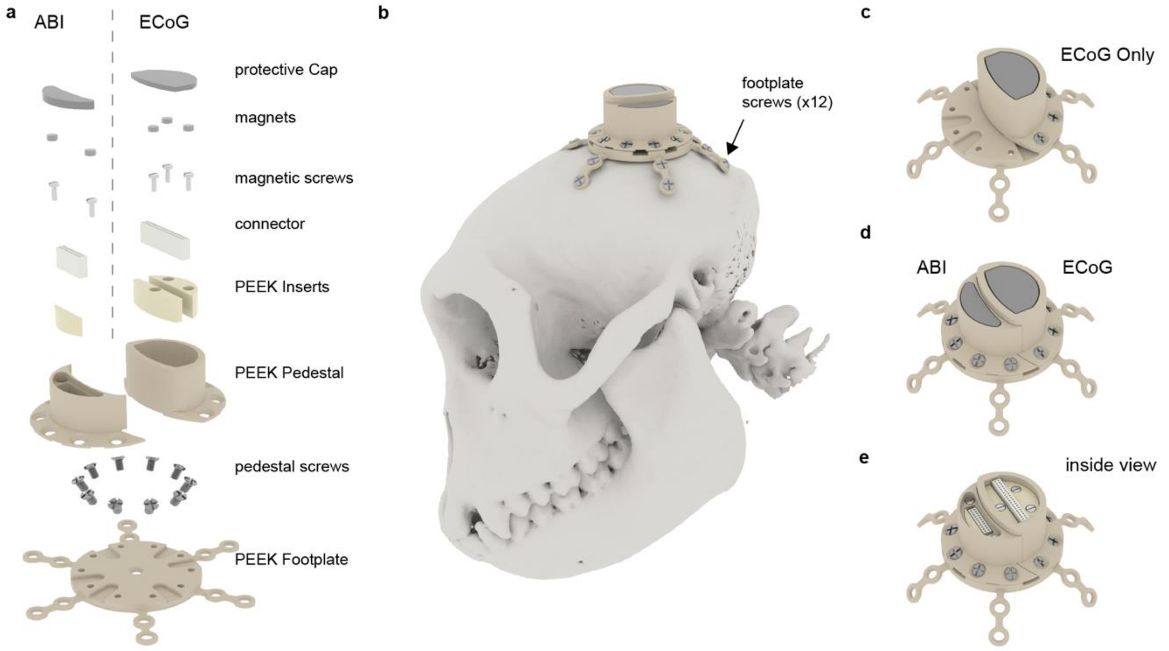

Supplementary Fig. 5 | Pedestal assembly. a. Exploded view of the system. **b.** System in place at the surface of the skull. **c.** ECoG pedestal only. **d.** ECoG and ABI pedestals. **e.** Inside view showing access to connectors.

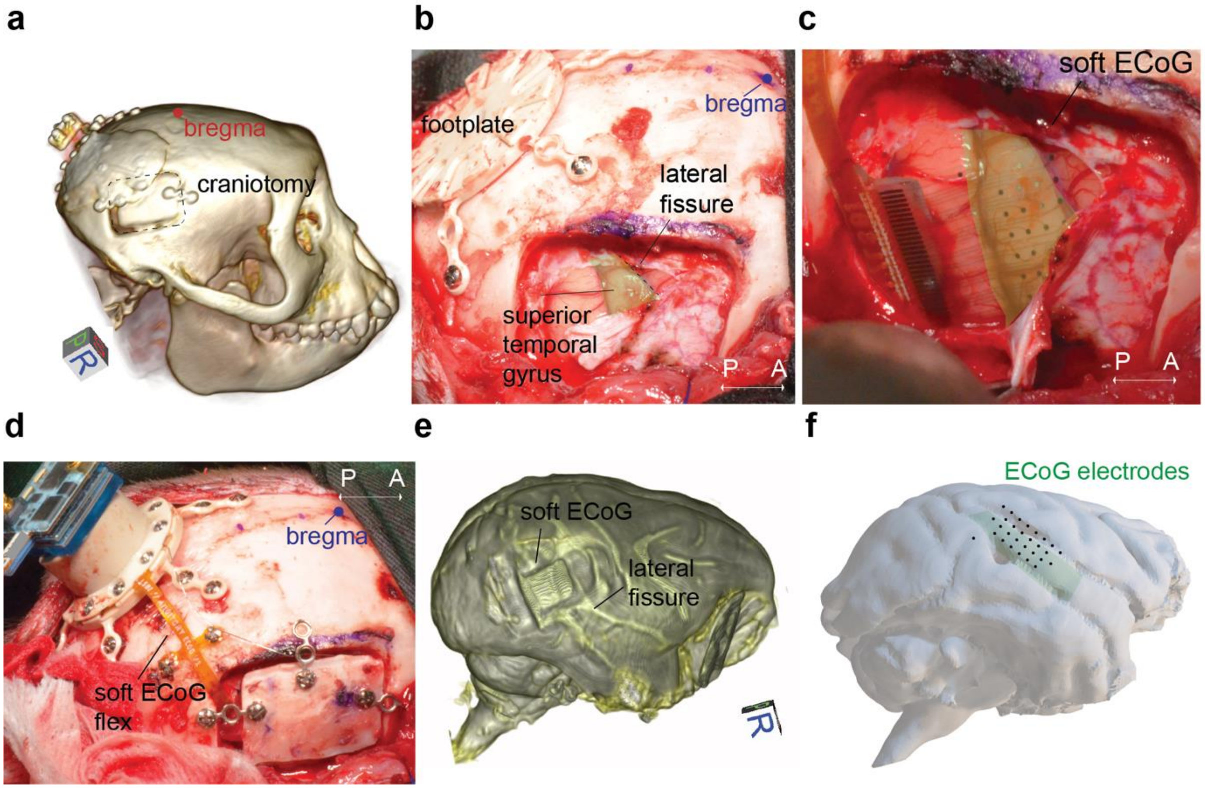

Supplementary Fig. 6 | Soft ECoG implantation procedure. Pictures show the procedure for monkey L (see Methods for details). **a.** 3D CT-scan reconstruction of the skull, with visible craniotomy and pedestal position relative to bregma. **b.** Image of the cranial window with the dura mater open, above the lateral fissure and superior temporal gyrus (outlined in light green). **c.** Soft ECoG subdural placement over the auditory cortex (outlined in light green). **d.** Picture of final placement, with bone flap and flex cable of the ECoG secured with titanium bridges. Wireless MCS system for recordings is also visible, as plugged to the ECoG through the omnetics connector. **e.** 3D MRI reconstruction showing ECoG placement relative to the full brain. **f.** Individual ECoG recording electrode position superimposed on the 3D MRI reconstruction. Approximate location of the auditory cortex is outlined in light green.

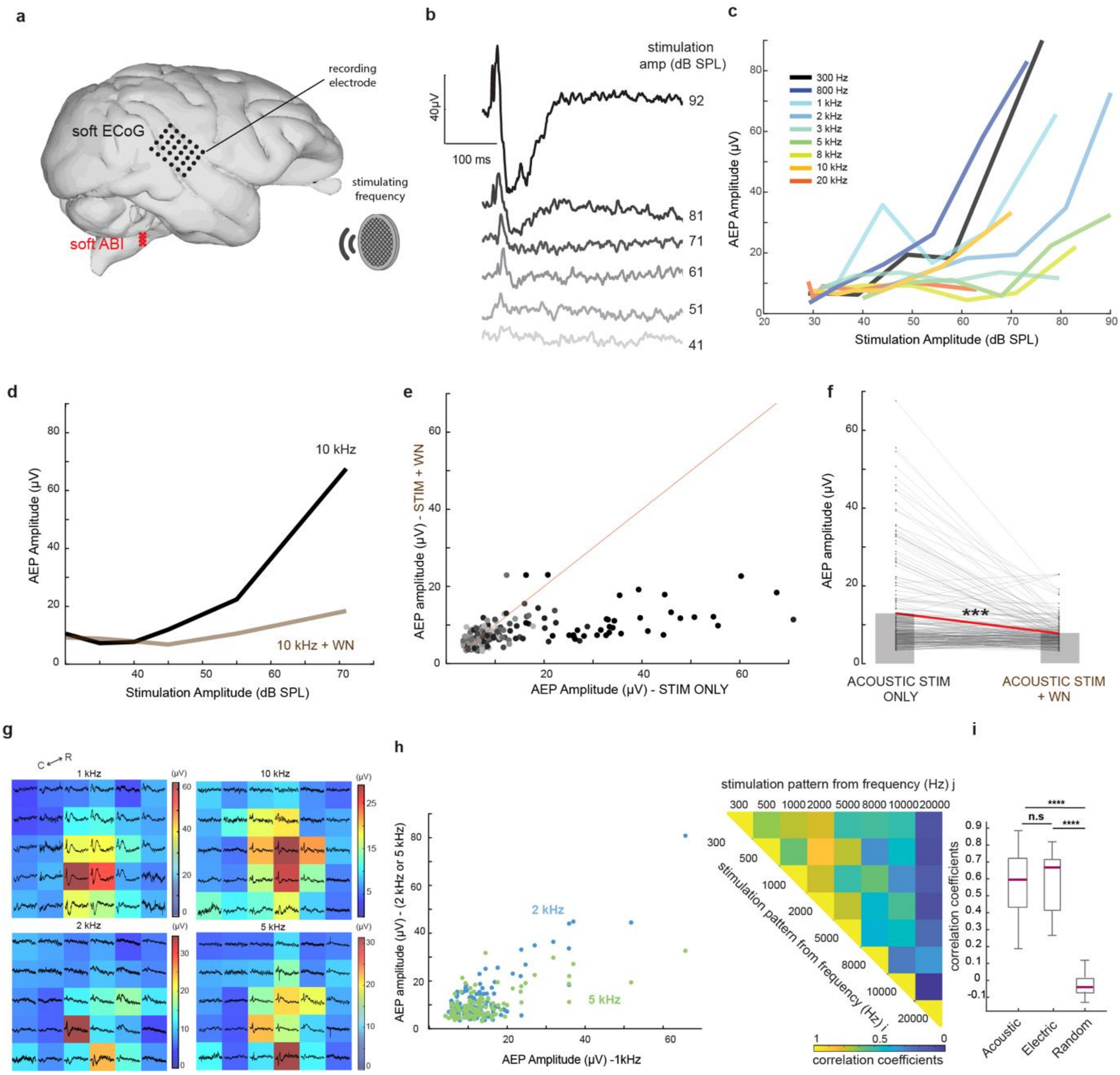
a
recording electrode
soft ECoG
stimulating frequency
soft ABI
b
stimulation amp (dB SPL)
40μV
100 ms
92
81
71
61
51
41
c
300 Hz
800 Hz
1 kHz
2 kHz
3 kHz
5 kHz
8 kHz
10 kHz
20 kHz
AEP Amplitude (μV)
Stimulation Amplitude (dB SPL)
d
10 kHz
10 kHz + WN
AEP Amplitude (μV)
Stimulation Amplitude (dB SPL)
e
AEP amplitude (μV) - STIM + WN
AEP Amplitude (μV) - STIM ONLY
f
AEP amplitude (μV)
***
ACOUSTIC STIM ONLY
ACOUSTIC STIM + WN
g
C R
1 kHz
10 kHz
2 kHz
5 kHz
(μV)
h
AEP amplitude (μV) - (2 kHz or 5 kHz)
AEP Amplitude (μV) -1kHz
2 kHz
5 kHz
stimulation pattern from frequency (Hz) j
300 500 1000 2000 5000 8000 10000 20000
stimulation pattern from frequency (Hz) i
correlation coefficients
i
n.s
****
****
correlation coefficients
Acoustic
Electric
Random

Supplementary Fig. 7 | Characterization of AEP cortical responses to acoustic stimulation. a. Schematic of the acoustic stimulation and recording devices at the surface of the auditory cortex. **b.** Evoked AEP responses recorded from ECoG (channel 1) for a 2kHz acoustic tone-burst. **c.** Recruitment curves for each tone frequency, when stimulated from 300 Hz to 20 kHz. **d.** Recruitment curves when stimulated with 10 kHz tone burst with (STIM+WN, brown curve) and without (STIM ONLY, black curve) acoustic white noise in the background. When acoustic white noise is also delivered AEPs amplitudes are decreased. **e.** Quantification of the white noise effect. Each dot represents one ECoG channel response (peak to peak amplitude) at a given stimulation amplitude (stimulation 10 kHz; same dB gray scale as (b)) with and without WN in the background. The distribution of the responses does not follow the x=y line. **f.** Mean of all responses is significantly different when WN is applied in the background (one tail paired t-test, $p<0.001$). **g.** Different acoustic frequencies evoke a distinct activity pattern at the surface of the auditory cortex. Colormaps represent the activity at the surface of the auditory cortex (AEPs peak to peak amplitude (μV)) depending on which frequency is played (at a given stimulation amplitude, here in this example 71 dB). Each trace represents the average over 500 ms. **h.** The graph shows a linear correlation between 1 and 2 kHz responses, while 1 and 5 kHz do not give correlated responses. Each dot represents a response from a recording electrode of the ECoG array at a given stimulation amplitude (ranging from 41 to 91 dB). Right: table of mean correlation coefficients from all ECoG recording channels, for each frequency pair. **i.** Box-plot showing comparison of correlation coefficients. Acoustic and electric correlation coefficients are not statistically different from each other (two-sided t-test, $p = 0.7701$) while they statistically differ from a random distribution (two-sided t-tests, $p < 0.0001$ ($p = 5.1530e-16$ and p5.1530e-16 and $p = 1.1294e-17$ respectively)). Panel a created using brain reconstruction from refs. 29, 32, and partly using BioRender.com.

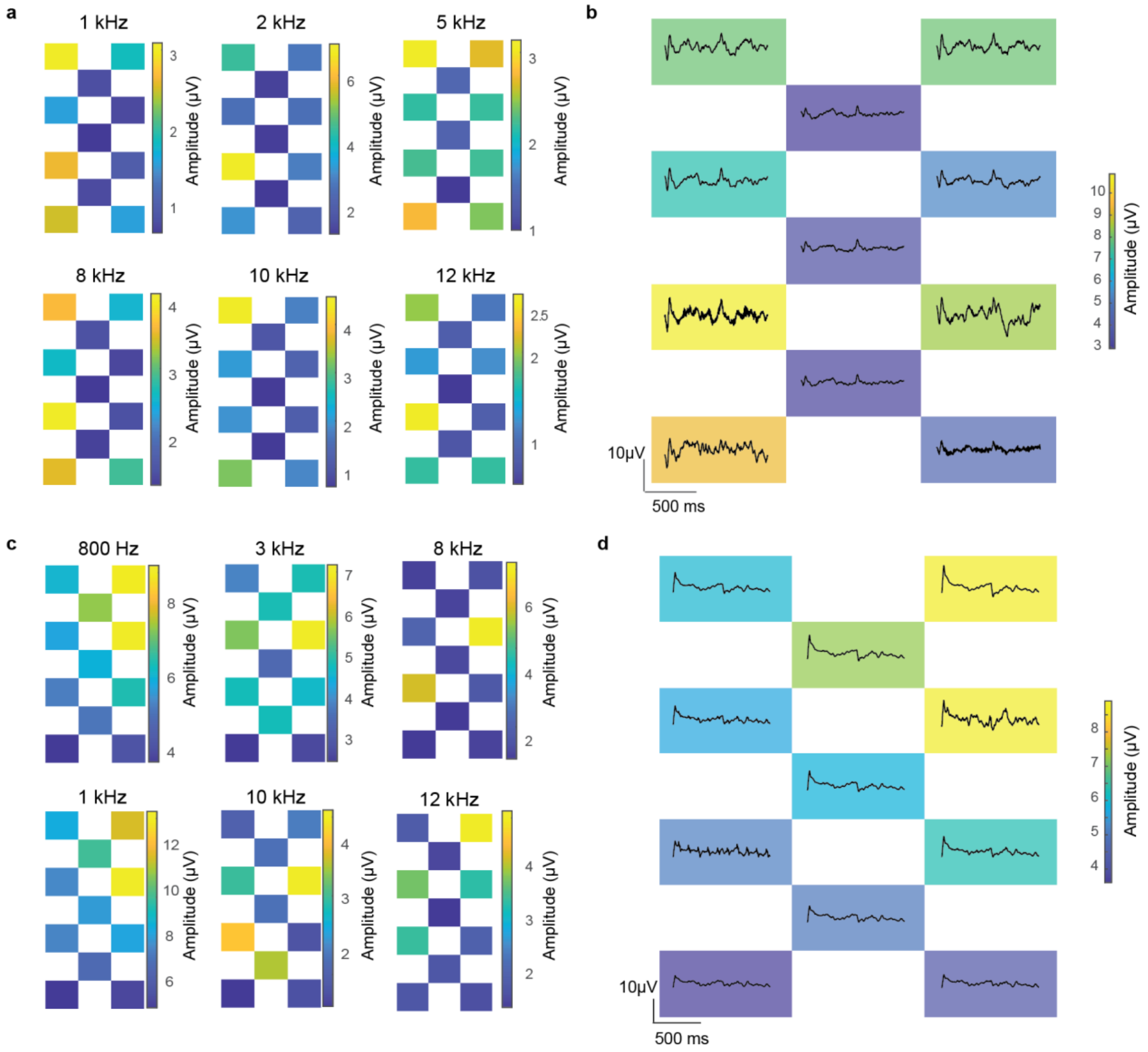

Supplementary Fig. 8 | Auditory evoked cochlear nucleus responses (AECNRs). a. AECNR amplitudes recorded from monkey L, under sedation, for different frequencies ranging from 1 to 12 kHz, at 71 dB SPL. Color maps highlight AECNR amplitudes. **b.** AECNR waveforms recorded from monkey L, in an awake condition, for 2 kHz at 87 dB SPL, acquired 5 months post ABI surgery. Signals, as well as color map representing AECNR amplitude as in a. are represented. **c.** AECNRs waveforms recorded from monkey G, under sedation, for different frequencies ranging from 800 Hz to 12 kHz, at 91-101 dB SPL. Color maps highlight AECNR amplitudes. **d.** Detailed view of the amplitude map and signals from the recordings shown in c., for monkey G, under sedation, with tone bursts of 800 Hz at 94 dB SPL.

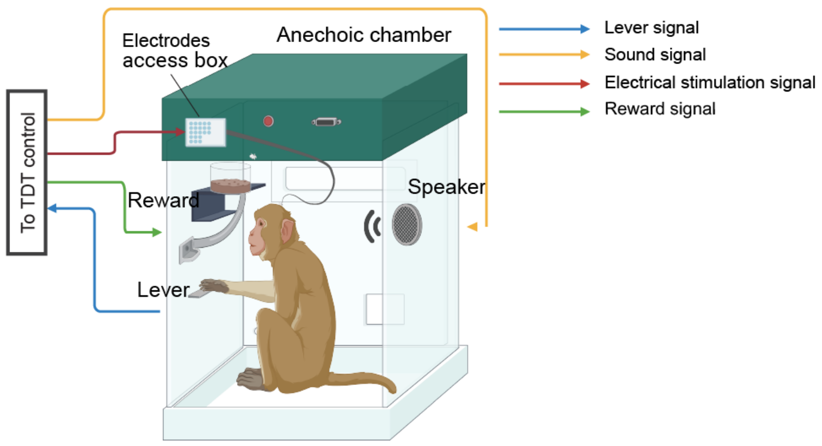

Supplementary Fig. 9 | Schematic of the behavioral set-up. The animal is sitting in a primate chair with arms free, inside a double-walled electrically shielded sound-proof chamber, where it can manipulate the lever and take rewards. Stimulation can be acoustic (through the speaker) and/or electric (through the soft ABI). Panel a created with BioRender.com.

z

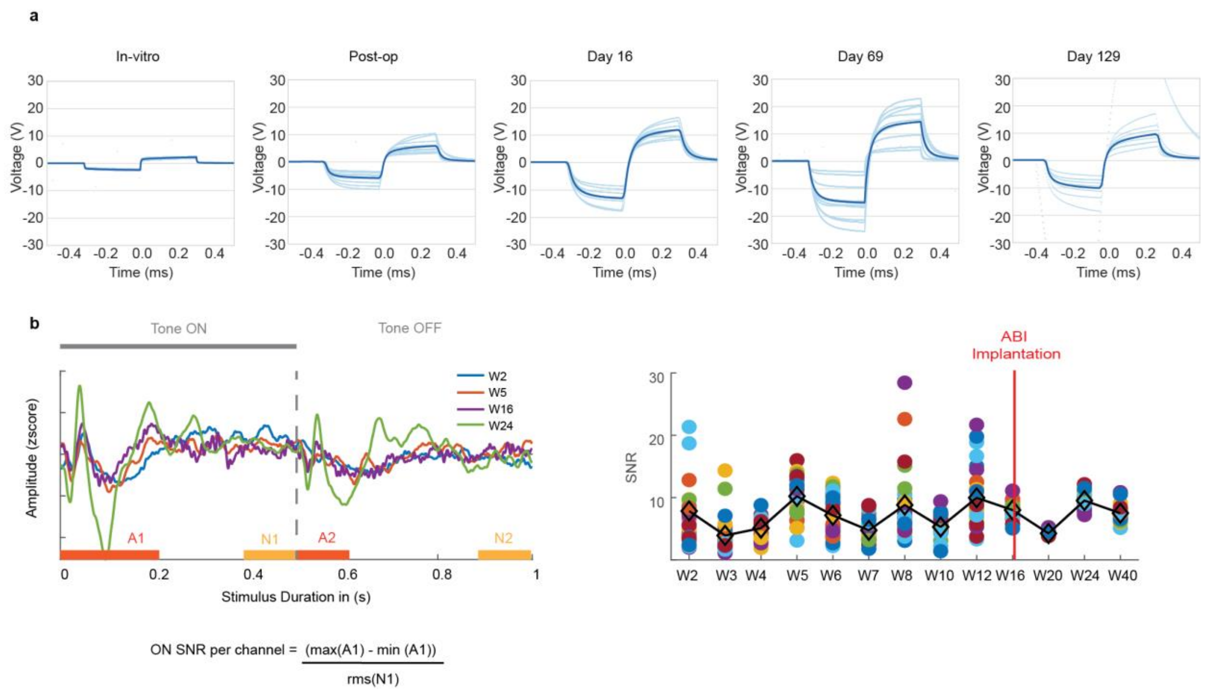

Supplementary Fig. 10 | Chronic stability ABI and ECoG devices. a. Voltage transients (VT) of the ABI devices, from in vitro post fabrication, up to 129 days (~4 months) in vivo. The data show an initial increase in access voltage, then stabilization. Each electrode's performance is illustrated in light blue, with the overall average presented in dark blue. Notably, on day 129, two electrodes were identified as broken and were consequently excluded from the averaging process. **b.** Overview of AEPs recorded over the duration of the experiment (40 weeks). Left plot shows typical AEP recordings (here, for 5 kHz tones), with onset and offset responses visible, taken at different time points (weeks (W) after ECoG implantation). Signal to noise ratio (SNR) is computed as the peak-to-peak amplitude of the zscored signal, within the first 200 ms following tone onset, divided by the rms of the noise in the last 100 ms (before tone offset). Right plot shows the computed SNR (here, for 10 kHz pure tones) of each recording electrode of the ECoG (black diamond represents the mean value) over multiple time points (x-axis label represents weeks (W) after ECoG implantation on which recordings were taken). ABI implantation surgery is highlighted in red. 5 months following ABI implantation, high SNR recordings were still possible from the ECoG.
